# ZNF217 promotes ovarian cancer progression by impacting multiple pivotal steps in the metastatic process

**DOI:** 10.1038/s41698-025-01153-8

**Published:** 2025-12-04

**Authors:** Kathryn C. Wardrup, Jessica Hoffman, Megha J. Pandya, Nithya Navarathna, Mohan E. Tulapurkar, Achuth Padmanabhan

**Affiliations:** 1 Department of Biological Sciences, University of Maryland Baltimore County, Baltimore, MD USA; 2 Division of Pulmonary and Critical Care Medicine, University of Maryland School of Medicine, Baltimore, MD USA; 3 University of Maryland Greenebaum Comprehensive Cancer Center, Baltimore, MD USA

**Keywords:** Ovarian cancer, Metastasis, Oncogenes

## Abstract

Ovarian cancer is characterized by aggressive metastasis, chemoresistance, and poor survival outcomes. Gaps in understanding of factors that drive these phenotypes have hindered the development of actionable therapeutic targets. We demonstrate that Zinc Finger Protein 217 (ZNF217) is a key pro-metastatic factor in ovarian cancer cells. ZNF217 overexpression dramatically increases proliferation, metastasis, and chemoresistance while its depletion impairs these phenotypes. Consistently, ZNF217 overexpression is associated with poor prognosis in both mouse models and ovarian cancer patients. Interestingly, ZNF217 induces metastatic phenotypes in fallopian tube cells, suggesting potential role in the transition of early-stage tumors to aggressive carcinoma. ZNF217’s oncogenic activity is dependent on its ability to bind DNA and alter multiple processes, including EMT, that are critical in driving different aspects of cancer progression. Thus, our data establishes ZNF217 as a potent oncogene in ovarian cancer cells that impacts multiple steps in the metastatic process and a potential therapeutic target.

## Introduction

Ovarian cancer is a lethal disease and a leading cause of cancer associated deaths among women in the United States^1^. Due to the vague nature of symptoms and the lack of reliable early biomarkers, over 70 percent of ovarian cancer patients have advanced metastatic disease at diagnosis^2^. Unfortunately, extant therapeutics and treatment strategies are ineffective at eliciting a durable response in these patients. As a result, the 5-year survival rate for women with metastatic ovarian cancer continues to be less than 30%^1^. To overcome this clinical challenge, it is important to identify key factors driving ovarian cancer metastasis and investigate their utility as a therapeutic target.

Zinc finger protein 217 (ZNF217) is a member of the C2H2 Kruppel-like zinc finger family of transcription factors and has been reported to play an important role in several biological processes such as cell proliferation, differentiation, development, cell motility, and programmed cell death^3^. The genomic locus harboring ZNF217 (20q13) is amplified in multiple human cancers^4^. Reports show that ZNF217 is deregulated in many cancers and contributes to disease progression^3,5^. In breast cancer cells ZNF217 induces ErbB3 expression, leading to an increase in PI-3/Akt signaling^6^. ZNF217 was shown to also mediate a matrix-stiffness and collagen density-induced increase in AKT activity and mammary epithelial cell proliferation^7^. In prostate cancer cells, ZNF217 decreased ferroportin concentration leading to increased intracellular iron retention, and iron-dependent cellular activities that enhanced tumor growth^8^. ZNF217 also induced proliferation, survival, migration, and stemness in human uterine leiomyosarcoma^9^. While ZNF217 levels have been shown to be elevated in ovarian cancer^10^, its role in ovarian cancer progression and metastasis has not been systematically investigated.

We demonstrate that ZNF217 functions as a potent oncogene in ovarian cancer cells. Using multiple clinically relevant in vitro and in vivo models, we show that ZNF217 drives ovarian cancer progression by impacting multiple important steps in the metastatic process. Our data reveal that ZNF217’s potential to function as an oncogene and drive metastasis in ovarian cancer cells is dependent on its ability to bind DNA and transcriptionally regulate genes. ZNF217 regulated genes control several key processes such as epithelial-to-mesenchymal transition (EMT) that are important in cancer progression. Importantly, we establish that ZNF217 overexpression alone is sufficient to induce oncogenic phenotypes in non-cancerous fallopian tube epithelial cells. Finally, we show that ZNF217 levels dictate the response of ovarian cancer cells to multiple chemotherapeutic drugs. Thus, our data establishes ZNF217 as a critical factor driving ovarian cancer progression and highlights its potential as an actionable therapeutic target to improve clinical outcome.

## Results

### ZNF217 overexpression is associated with poor prognosis in ovarian cancer

**Fig. 1:**
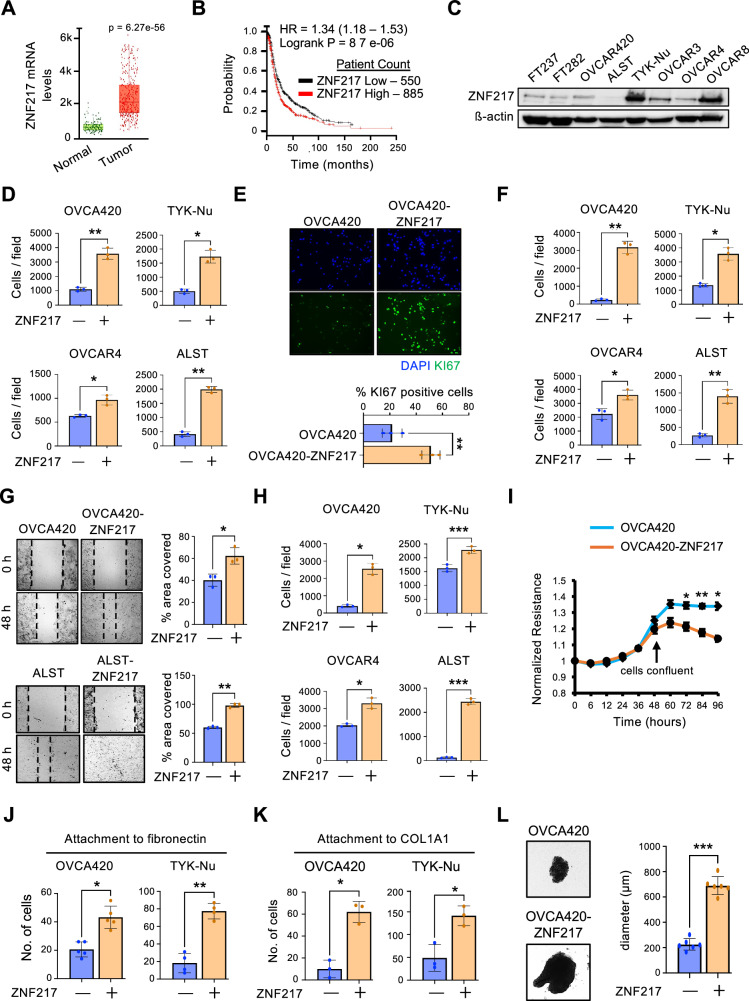
ZNF217 overexpression promotes ovarian cancer is associated with poor prognosis in ovarian cancer. **A** ZNF217 mRNA is elevated in ovarian tumor tissue as compared to normal (non-cancerous) ovarian tissue (n = 744, p = 2.13e-11). **B** High levels of ZNF217 are associated with a decrease in overall survival (n = 1435, logrank p = 8.7e-06). **C** Western blot showing ZNF217 levels in human fallopian tube and ovarian cancer cell lines. **D** ZNF217 overexpression increases proliferation in ovarian cancer cells (n = 3). **E** OVCA420-ZNF217 cells have elevated Ki67 staining compared to OVCA420 cells (n = 3). **F** ZNF217 increases migration of ovarian cancer cells in transwell assay (n = 3). **G** ZNF217 increases migration of ovarian cancer cells in wound healing assay (n = 3). **H** ZNF217 increases the ability of ovarian cancer cells to invade through Matrigel (n = 3). **I** Electric Cell-Substrate Impedance Sensing (ECIS) assay show that ZNF217 overexpressing OVCA420 cells display an increase in resistance (R) measured at 1000 Hz (n = 3). **J** ZNF217 increases attachment of ovarian cancer cells to fibronectin (n = 3). **K** ZNF217 increased attachment of ovarian cancer cells to collagen (n = 3). **L** ZNF217 overexpressing OVCA420 cells form larger spheroids (n = 6).

The genomic locus harboring *ZNF217*, 20q13.2, is amplified in 31% ovarian cancer cases, 40% of which have increased ZNF217 mRNA expression^11^. Publicly available gene expression datasets show that ZNF217 expression is significantly elevated in several cancers, including ovarian cancer (Fig. 1A, S1A, and S1B). Elevated ZNF217 protein was reported in 60% of cases in a study that analyzed tumors from 44 ovarian cancer patients by immunohistochemistry^12^. Importantly, elevated ZNF217 in these patients was associated with poor prognosis^12^. Further, ZNF217 amplification also correlates with increased lymph node metastasis in ovarian clear cell carcinomas^13^. To ascertain potential correlation between ZNF217 and clinical outcome in ovarian cancer patients, we analyzed data from 1435 ovarian cancer patients that is publicly available through KmPlotter^14,15^. Elevated ZNF217 in this dataset correlated with poor progression-free survival in patients (Fig. 1B). These data show that ZNF217 is overexpressed in ovarian cancer and is associated poor clinical outcome. Despite these compelling data, ZNF217’s precise role in ovarian cancer remains poorly understood.

### ZNF217 overexpression in ovarian cancer cells promotes oncogenic phenotypes in vitro

Comparison of basal ZNF217 levels across six different ovarian cancer cell lines and two immortalized fallopian tube cell lines revealed higher ZNF217 levels in ovarian cancer cells (Fig. 1C). Among ovarian cancer cells, OVCA420, OVCAR4, and ALST cells expressed lower ZNF217 levels compared to TYK-Nu cells. To determine how ectopic ZNF217 expression impacts oncogenic phenotypes, we stably expressed ZNF217 in these cells (Fig. S1C). In vitro assays show that ZNF217 overexpression causes a dramatic increase in proliferation as measured by crystal violet staining (Fig. 1D and S1D). The observed increase in the proliferation of ZNF217 overexpressing cells in culture was supported by a corresponding increase in Ki67 staining in OVCA420-ZNF217 cells (Fig. 1E) as well as a quantitative increase in proliferation as determined by WST-1 proliferation assay (Fig S1E). We also noticed that the number of cells in the G2-phase of the cell cycle was higher in ZNF217 overexpressing OVCA420 cells (Fig. S1F). In addition to stimulating proliferation, ZNF217 expression also enhanced cellular migration (Fig. 1F, G and S1G) and invasion through matrigel (Fig. 1H and S1H). As ZNF217 overexpression impacted both proliferation and migration, we wanted to ascertain that the effect on cell migration is not impacted by differences in cell proliferation. To address this concern, we measured differences in cell migration in the presence of mitomycin C (MMC), an agent that causes cell cycle arrest. ZNF217 overexpression in OVCA420 cells resulted in increased migration even in the presence of MMC (Fig. S1I). Early stages in metastasis involve activation of pathways that result in loosening of cell-cell junctions. To quantitatively measure ZNF217’s impact on tight junctions in real time, we performed electric cell-substrate impedance sensing (ECIS) assay using parental and ZNF217 overexpressing OVCA420 cells. The resistance across a confluent OVCA420-ZNF217 monolayer was consistently lower suggesting that these cells have weaker tight junctions (Fig. 1I and S1J), which further supports ZNF217’s ability to promote ovarian cancer cell migration. The ability to attach to extracellular matrix (ECM) is critical for metastatic cancer cells to invade neighboring tissues. A significant increase in ovarian cancer cell’s ability to attach to ECM proteins such as fibronectin (Fig. 1J and S1K) and collagen type I alpha 1 chain (COL1A1) (Fig. 1K and S1K) was observed upon ZNF217 overexpression. During metastasis, ovarian cancer cells from the primary tumor are shed into the peritoneal cavity. The ability of these cells to aggregate and form multicellular spheroids has been shown to be critical for successful metastasis through the peritoneal cavity^16,17^. Interestingly, ZNF217 overexpression increased the size of the spheroids formed by OVCA420 cells in suspension (Fig. 1L). Collectively, our data demonstrates that ZNF217 overexpression impacts several critical steps involved in ovarian cancer progression and metastasis.

In addition to differences in ZNF217 levels, the cell lines used in these experiments also differ in their p53 status. While ALST has wild-type p53, TYK-Nu, OVCA420 and OVCAR4 harbor p53-R175H, p53-R273H, and p53-L130V mutations respectively. Mutations in p53 are observed in over 96% high-grade serous ovarian cancers and play an important role in disease progression and therapeutic resistance^18–20^. Among p53 mutations, p53-R175H and p53-R273H are the most frequent in ovarian cancer and are thus clinically most relevant. ZNF217 promoted oncogenic phenotypes in all the cell lines we have tested so far suggesting that ZNF217 can impact tumor progression in a broad spectrum of ovarian cancer patients with tumors that differ in their p53 status.

### ZNF217 depletion inhibits ovarian cancer cell proliferation and metastatic potential in vitro

**Fig. 2:**
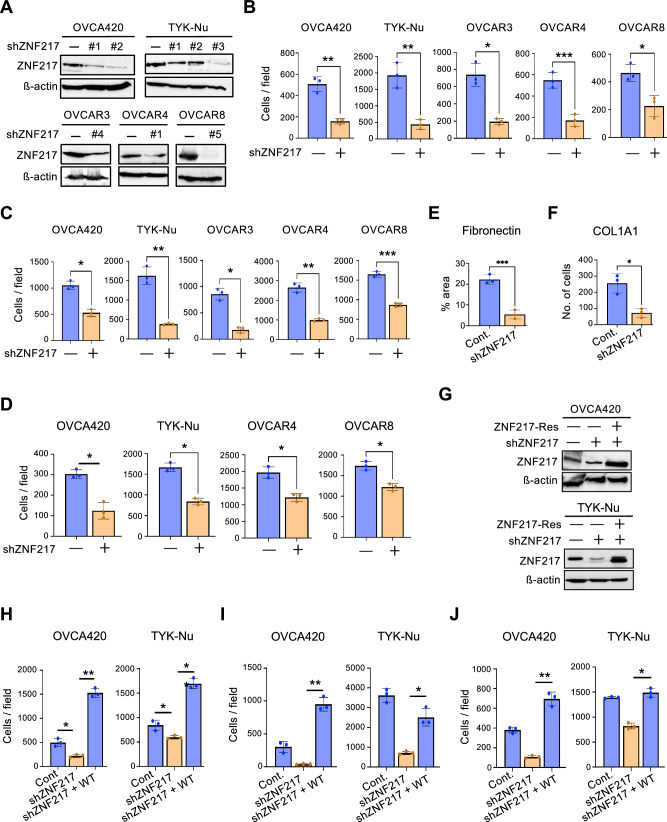
ZNF217 depletion impairs ovarian cancer cell proliferation and metastatic potential in vitro. **A** Western blot showing stable shRNA mediated ZNF217 knockdown in ovarian cancer cells. **B** ZNF217 knockdown in OVCA420 and TYK-Nu cells causes a reduction in cell proliferation (n = 3). **C** Transwell migration assay show that ZNF217 depletion reduces ovarian cancer cell migration (n = 3). **D** ZNF217 knockdown decreases the ability of ovarian cancer cells to invade through Matrigel (n = 3). **E** ZNF217 knockdown decreases attachment of OVCA420 cells to fibronectin coated plates (n = 3). **F** ZNF217 knockdown decreases attachment of OVCA420 cells to collagen coated surface (n = 3). **G** Western blot confirming successful re-expression of shRNA resistant ZNF217 in OVCA420 and TYK-Nu ZNF217 knockdown cells. **H** Restoration of ZNF217 expression using an shRNA-resistant form of ZNF217 rescues defects in cell proliferation in OVCA420 and TYK-Nu cells (n = 3). **I** Restoration of ZNF217 expression using an shRNA-resistant form of ZNF217 rescues defects in cell migration in OVCA420 and TYK-Nu cells (n = 3). **J** Restoration of ZNF217 expression using an shRNA-resistant form of ZNF217 rescues defects in OVCA420 and TYK-Nu invasion through the Matrigel (n = 3).

To confirm the oncogenic phenotypes observed upon ZNF217 overexpression and to determine the utility of ZNF217 as a therapeutic target, we stably expressed shRNA targeting different regions of the ZNF217 mRNA in five different human ovarian cancer cell lines: OVCA420, TYK-Nu, OVCAR4, OVCAR3, and OVCAR8. Stable ZNF217 knockdown in these cells was confirmed by western blot (Fig. 2A). ZNF217 depletion significantly reduced the ability of these cells to proliferate as measured by both crystal violet staining (Fig. 2B and S2A) and WST-1 assay (Figure S2B). The ability of ovarian cancer cells to migrate (Fig. 2C and S2C) and invade through matrigel (Fig. 2D and S2D) were also negatively impacted by ZNF217 depletion. Further, ZNF217 knockdown impaired the attachment of OVCA420 cells to ECM proteins such as fibronectin (Fig. 2E) and COL1A1 (Fig. 2F). These data from multiple ovarian cancer cell lines that differ in their p53 status provide compelling evidence to support ZNF217’s function as a critical oncogene that regulates ovarian cancer cell proliferation and metastatic potential.

To ascertain the specificity of ZNF217 shRNA, we tested if an shRNA resistant ZNF217 could rescue the phenotypes observed upon ZNF217 depletion. To this end, we stably expressed ZNF217 in OVCA420 and TYK-Nu cells expressing a shRNA targeting the ZNF217 3’UTR (Fig. 2G). The shRNA-resistant ZNF217 rescued the ability of these cells to proliferate (Fig. 2H and S2E), migrate (Fig. 2I and S2F), and invade through matrigel (Fig. 2J and S2G). Consistent with higher ZNF217 levels in the stable cells that express the shRNA-resistant ZNF217 (Fig. 2G), we observed that these cells exhibited higher proliferative and metastatic potential in the in vitro assays compared to control cells. These data confirm that ZNF217 depletion decreases proliferation and metastatic potential of ovarian cancer cells and suggest that the oncogenic effect of ZNF217 on ovarian cancer cells is likely to be dose dependent.

### ZNF217’s oncogenic potential is dependent on its ability to bind DNA

**Fig. 3:**
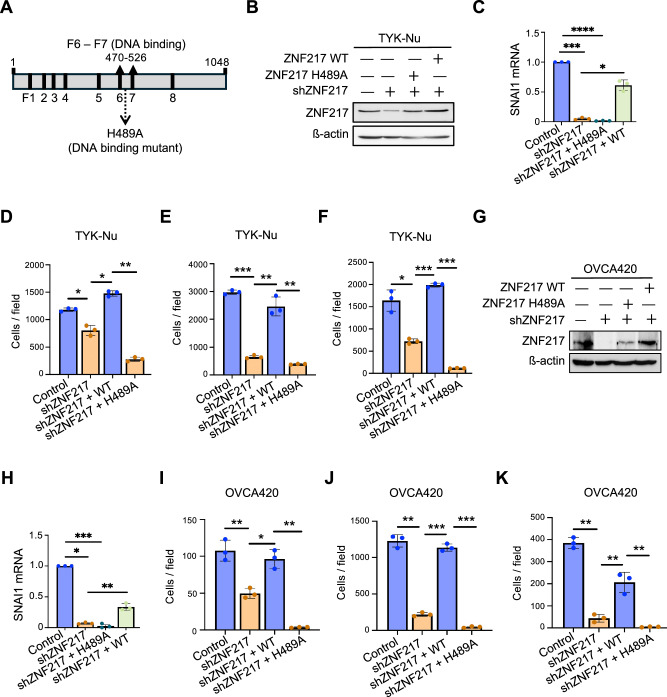
Oncogenic activity of ZNF217 in ovarian cancer cells is dependent on its ability to bind DNA. **A** Schematic representation of ZNF217’s eight C2H2 Zn-finger domains with the position of the H489A mutation that compromises the DNA binding ability of ZNF217. **B** Western blot confirming stable expression of shRNA resistant ZNF217-WT and ZNF217-H489A in TYK-Nu cells that stably express ZNF217 shRNA. **C** RT-qPCR data show that unlike wild type (WT) ZNF2117, ZNF217-H489A expression does not rescue the expression of SNAI1 in ZNF217 depleted TYK-Nu cells (n = 3). **D** Proliferation defects due to ZNF217 depletion in TYK-Nu cells is be rescued by an shRNA-resistant ZNF217-WT expression, but not ZNF217-H489A (n = 3). **E** Transwell migration assay showing that shRNA-resistant ZNF217-WT, but not ZNF217-H489A, rescues defects in TYK-Nu migration induced upon ZNF217 knockdown (n = 3). **F** Unlike ZNF217-WT, ZNF217-H489A mutant does not rescue the impact of ZNF217 depletion on the ability of TYK-Nu cells to invade through the Matrigel (n = 3). **G** Western blot confirming stable expression of shRNA resistant ZNF217-WT and ZNF217-H489A in OVCA420 cells that stably express ZNF217 shRNA. **H** RT-qPCR data show that unlike WT ZNF217, ZNF217-H489A expression does not rescue the expression of SNAI1 in ZNF217 depleted OVCA420 cells (n = 3). **I** Proliferation defects due to ZNF217 depletion in OVCA420 cells can be rescued by an shRNA-resistant ZNF217-WT expression, but not ZNF217-H489A (n = 3). **J** shRNA-resistant ZNF217-WT, but not ZNF217-H489A, rescues defects in OVCA420 migration induced upon ZNF217 knockdown (n = 3). **K** Unlike ZNF217-WT, ZNF217-H489A mutant does not rescue the impact of ZNF217 depletion on the ability of OVCA420 cells to invade through the Matrigel (n = 3).

While transcription factors mediate biological effects primarily by altering expression of downstream genes, they can also impact cellular processes through protein-protein interactions that are independent of their transcriptional activity. Previous studies discovered that the Zn-finger domains 6 and 7 within ZNF217 are critical in mediating interaction with DNA and identified a single amino acid mutation, H489A, that impaired DNA binding by disrupting ZNF217’s ability to bind zinc (Fig. 3A)^21^. We hypothesized that, if the DNA-binding ability of ZNF217 is critical for its oncogenic activity, ZNF217-H489A mutant will be deficient in rescuing the phenotypes observed upon ZNF217 knockdown. To test this, we stably expressed ZNF217-H489A mutant in TYK-Nu cells expressing ZNF217 shRNA (Fig. 3B). ZNF217 has been previously shown to bind to the promoter of SNAI1 directly and increase its expression^22^. ZNF217 knockdown caused a decrease in SNAI1 expression, which was rescued upon expression of shRNA-resistant wild-type ZNF217 (Fig. 3C). Consistent with its impaired ability to bind DNA, ZNF217-H489A did not rescue SNAI1 expression (Fig. 3C). Interestingly, unlike wild-type ZNF217, ZNF217-H489A did not rescue the effect of ZNF217 knockdown on cell proliferation (Fig. 3D and S3A), migration (Fig. 3E and S3B), and invasion (Fig. 3F and S3C) in TYK-Nu cells. To confirm that our results are not a cell line specific artifact, we stably expressed the shRNA-resistant ZNF217-H489A mutant in OVCA420 cells expressing ZNF217 shRNA (Fig. 3G). Again, unlike wild-type ZNF217, ZNF217-H489A mutant did not rescue SNAI1 expression in shZNF217 expressing OVCA420 cells confirming that this mutant is defective in DNA binding (Fig. 3H). As with TYK-Nu cells, we observed that the ZNF217-H489A mutant was deficient in rescuing the effect of ZNF217 knockdown on proliferation (Fig. 3I and S3D), migration (Fig. 3J and S3E), and invasion (Fig. 3K and S3F) in OVCA420 cells. These data establish that ZNF217’s ability to bind DNA and regulate gene expression is critical for its ability to function as an oncogene.

### ZNF217 drives epithelial-mesenchymal transition in ovarian cancer cells

**Fig. 4:**
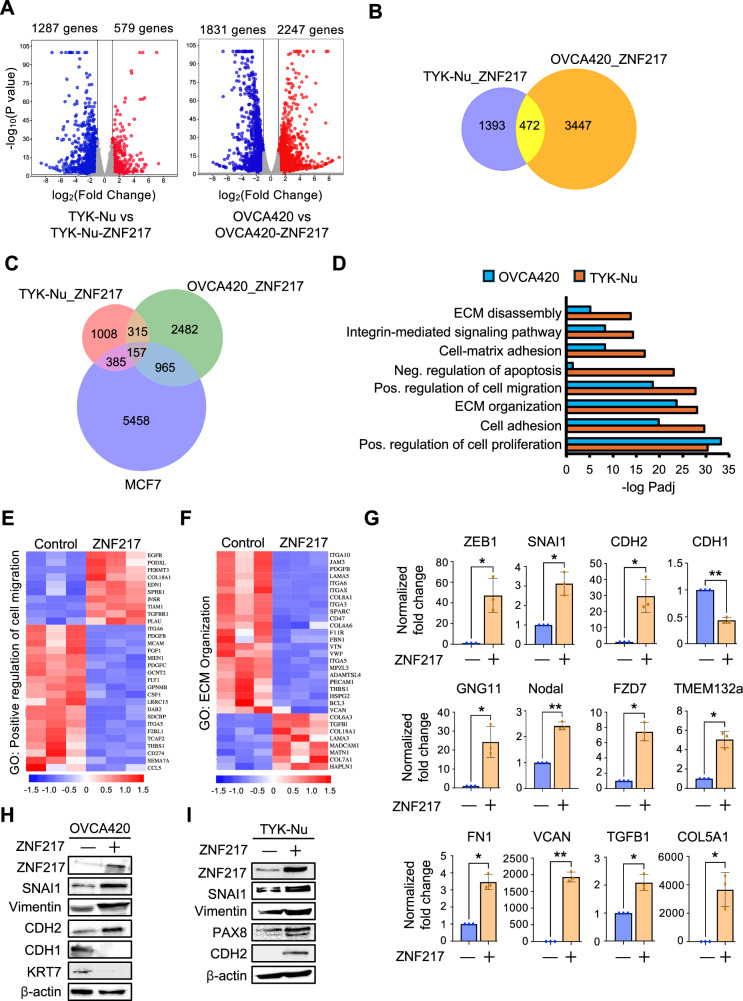
ZNF217 overexpression results in pro-metastatic transcriptomic changes in ovarian cancer cells. **A** Volcano plot showing differentially regulated genes in ZNF217 overexpressing TYK-Nu and OVCA420 cells (n = 3; p-value < 0.05; log2 fold change >1). **B** Venn diagram showing number of differentially regulated genes that were unique and shared between TYK-Nu and OVCA420 cells. **C** Venn diagram showing overlap between ZNF217 regulated genes in TYK-Nu and OVCA420 cells with CHIP-seq data from MCF7 cells. **D** Gene ontology terms that are significantly enriched in the differentially expressed gene sets in OVCA420 and TYK-Nu cells. **E** Heatmap showing differentially regulated genes related to regulation of cell migration. **F** Heat map showing differentially regulated genes related to ECM organization. **G** RT-qPCR analyses of OVCA420 and OVCA420-ZNF217 cells reveals differential regulation of EMT-associated genes (n = 3). **H** Western blot show EMT markers are altered upon ZNF217 overexpression in OVCA420. **I** Western blot show EMT markers are altered upon ZNF217 overexpression in TYK-Nu cells.

As ZNF217’s ability to bind DNA is critical for its oncogenic function, we determined the effect of overexpressing ZNF217 on the ovarian cancer cell’s transcriptome. RNAseq revealed that 1866 and 4078 genes were differentially regulated upon ZNF217 overexpression in TYK-Nu and OVCA420 cells respectively (Fig. 4A, Table S1, and Table S2). While ZNF217 has been previously suggested to function mostly as a transcriptional repressor^23–25^, our data reveal that several genes are upregulated upon ZNF217 overexpression. Specifically, we observe 2247 genes are upregulated compared to 1831 genes being repressed upon ZNF217 overexpression in OVCA420 cells (Fig. 4A). In comparison to TYK-Nu, we observe a greater number of genes to be differentially expressed in OVCA420 upon ZNF217 overexpression possibly because TYK-Nu has a higher basal ZNF217 level (Fig. 1C). Among the differentially regulated genes, 472 genes were shared between TYK-Nu and OVCA420 cells, suggesting that they might be critical for the observed ZNF217-induced oncogenic phenotypes (Fig. 4B and Table S3). To determine the differentially expressed genes in TYK-Nu and OVCA420 cells that are potential direct ZNF217 targets, we compared them with a previously identified list of ZNF217 direct targets in MCF-7 cells^26^. Our analysis shows that 1122 of the 4078 differentially regulated genes in OVCA420 and 542 of the 1866 differentially regulated genes in TYK-Nu could potentially be direct ZNF217 targets (Fig. 4C and Table S4). Consistent with ZNF217’s ability to function as a potent oncogene, gene ontology analysis revealed that key processes such as cell proliferation, negative regulation of apoptosis, cell migration, cell adhesion, ECM organization, and cell-matrix adhesion are differentially regulated upon ZNF217 overexpression in both TYK-Nu and OVCA420 cells (Fig. 4D–E F and S4A). As increased migratory and invasive potential is dependent on the acquisition of a more mesenchymal state, we determined if ZNF217 expression induces EMT in ovarian cancer cells. RT-qPCR analysis revealed that expression of several EMT-associated genes is altered in OVCA420 cells upon ZNF217 overexpression (Fig. 4G). This includes established EMT regulators such as ZEB1 and SNAI1, as well as cell adhesion molecules such as CDH1 and CDH2. Consistent with the gene expression data, western blot showed an increase in mesenchymal markers such as SNAI1, vimentin, N-cadherin (CDH2), and a concomitant decrease in epithelial markers such as E-cadherin (CDH1) and cytokeratin 7 (KRT7) upon ZNF217 overexpression (Fig. 4H, I, and S4B). These data raise the possibility that ZNF217 may function as a master transcription factor that regulates EMT, much like Zeb1 and SNAI1. Taken together, our data suggests that ZNF217 overexpression induces largescale gene expression changes in ovarian cancer cells, which increases their proliferation, suppresses apoptosis, promotes the acquisition of a more mesenchymal state, and induces extracellular matrix reorganization. By regulating a broad range of critical processes intrinsic to a cancer cell, ZNF217 can impact multiple stages of tumor progression.

### ZNF217 promotes ovarian cancer metastasis in vivo

**Fig. 5:**
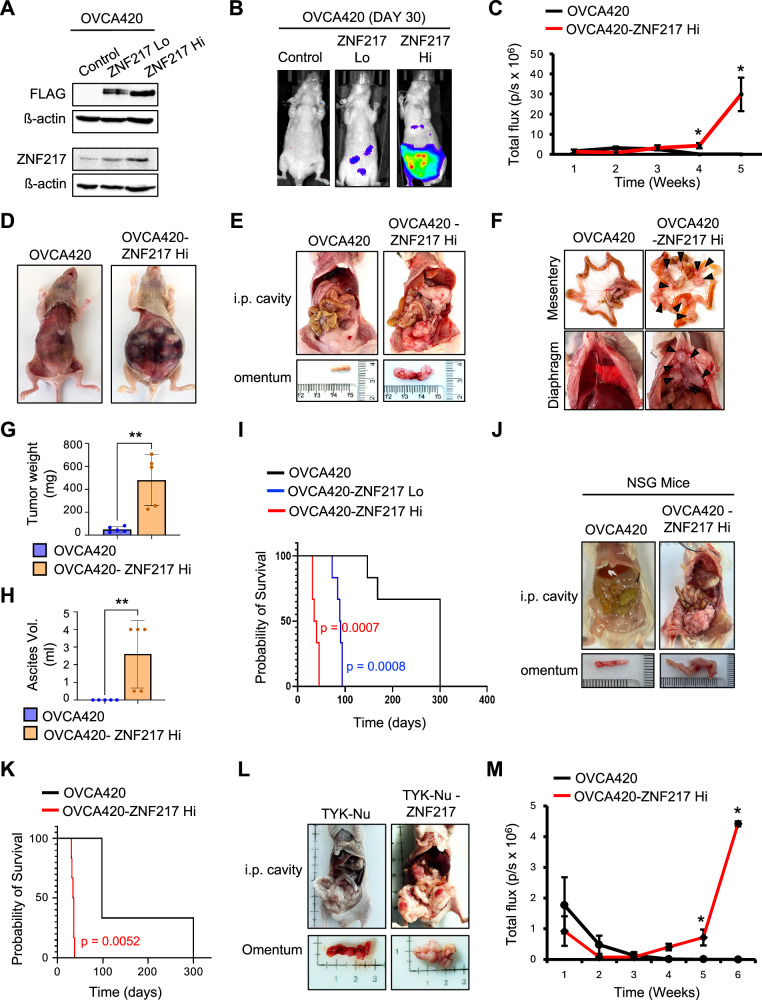
ZNF217 promotes ovarian cancer metastasis in vivo. **A** Western blot confirming OVCA420-ZNF217-Lo and OVCA420-ZNF217-Hi cells. **B** Luciferase signal on day 30 post i.p injection shows that ZNF217 promotes metastasis in Foxn1 nude mice in a dose-dependent manner. **C** In vivo imaging show that OVCA420-ZNF217-Hi cells form metastatic tumors at a faster rate than control cells in foxn1 nude mice upon i.p injection (n = 7 mice/group). Tumor progression in OVCA420-ZNF217-Hi cells is also faster compared to OVCA420-ZNF217-Lo cells (Fig. S6C). **D** Female Foxn1 nude mice i.p injected with OVCA420-ZNF217-Hi cells exhibit enlarged peritoneal cavity and accumulation of ascites. Enlargement of peritoneal cavity is not upon injection of OVCA420-FUCRW (control) cells. **E** OVCA420-ZNF217-Hi cells form large tumors in the i.p cavity. No visible tumors were detected in mice injected with OVCA420-FUCRW cells. **F** Increased metastatic tumor burden is observed on the mesentery and diaphragm in mice i.p injected with OVCA420-ZNF217-Hi cells. **G** Quantification showing significantly higher tumor burden on the omentum in mice containing ZNF217-high tumors (n = 5 mice/group). **H** Quantification showing significantly more ascites in mice containing ZNF217-Hi tumors (n = 5 mice/group). **I** Kaplan-Meier curve shows that ZNF217 exerts a dose dependent effect on survival in an i.p model of metastatic ovarian cancer (n = 6 mice/group). **J** ZNF217 promotes aggressive metastatic tumors in the immunodeficient NSG mice (n = 6 mice/group). Representative images are shown. **K** Kaplan–Meier curve shows that ZNF217 overexpression causes a decrease in survival in an i.p model of metastatic ovarian cancer in NSG mice. n = 6 mice/group; p = 0.0052. **L** ZNF217 overexpression increases tumor forming ability of TYK-Nu cells in Foxn1 nude mice (i.p model) (n = 6 mice/group). Representative images are shown. **M** ZNF217 overexpression increases tumor forming ability of OVCA420 cells in Foxn1 nude mice in an ovarian intra-bursal injection model (n = 3 mice/group).

To determine how ZNF217’s oncogenic effects translate to an in vivo setting and to ascertain if ZNF217 exerts a dose-dependent effect on tumor progression, we derived luciferase-tagged OVCA420 cells that stably expresses low (ZNF217-Lo) and high (ZNF217-Hi) levels of FLAG-tagged ZNF217 (Fig. 5A). These cells were injected intraperitoneally (i.p) into immunodeficient female athymic Foxn1 nude mice. The i.p injection model closely mimics the physiological route and microenvironment through which ovarian cancer cells metastasize and is thus a clinically relevant and widely used model to study ovarian cancer metastasis. Non-invasive imaging revealed that ZNF217 overexpression dramatically increased the ability of OVCA420 cells to form metastatic tumors in this model (Fig. 5B, C, S5A, and S5B). Notably, ZNF217-Hi cells were more aggressive in forming tumors compared to ZNF217-Lo cells suggesting that ZNF217 exerts a dose dependent effect on tumor progression (Fig. 5B, C, S5A, S5B, and S5C). Mice injected with ZNF217-Hi OVCA420 cells had a significantly larger tumor burden within the peritoneal space compared to mice injected with control cells (Fig. 5D, E). OVCA420-ZNF217 cells formed large tumors at common ovarian cancer metastatic sites such as the omentum, mesentery and diaphragm (Fig. 5E–FG, S5D, S5E, and S5F). These sites either had small tumors or none in mice injected with control cells (Fig. 5–F, G, S5D, S5E, and S5F). Accumulation of ascites fluid is a hallmark of advanced ovarian cancer. Consistent with ZNF217’s ability to promote aggressive metastasis and increase tumor burden, ascites fluid accumulated in mice bearing ZNF217 overexpressing tumors (Fig. 5H). In comparison, mice injected with control OVCA420 cells exhibited no accumulation of ascites fluid (Fig. 5H). Consistent with ZNF217’s ability to drive aggressive metastatic tumor formation, i.p injection of ZNF217 overexpressing cells resulted in a striking decrease in survival in Foxn1 nude mice (Fig. 5I). Consistent with its effect on tumor progression, ZNF217 exerted a dose-dependent effect on survival (Fig. 5I). Mice bearing ZNF217-Hi tumors had the shortest survival time (Fig. 5I). The median survival was 37 days, 89.5 days and 300 days for mice bearing ZNF217-Hi, ZNF217-Lo, and control tumors respectively (Fig. 5I). To confirm that ZNF217’s effect on ovarian cancer metastasis and survival is not dependent on the strain of immunodeficient mice used, we repeated these studies using a different strain of immunodeficient mice, the NOD *scid* gamma (NSG) mice. As in Foxn1 mice, in an NSG i.p injection model using OVCA420 cells, ZNF217 overexpression resulted in increased metastasis (Fig. 5J), higher tumor burden (Fig. 5J), and decreased survival (Fig. 5K). In vitro studies showed that ZNF217 exerted similar pro-tumor effects on different ovarian cancer cells that differ in their p53-status and ZNF217 expression levels. To ascertain if this is true in vivo, we performed in vivo experiments using control and ZNF217 overexpressing TYK-Nu cells using the i.p injection model in female Foxn1 nude mice. As with OVCA420 cells, ZNF217 overexpression in TYK-Nu cells also resulted in increased metastasis and tumor burden in Foxn1 nude mice (Fig. 5L).

The i.p injection model recapitulates events in ovarian cancer metastasis post-shedding of cancer cells into the peritoneal cavity. Prior to this stage, metastatic cancer cells must acquire the ability to form early-stage tumors on the fallopian tube and ovary. To determine if ZNF217 can impact early stages in metastasis, we injected luciferase-tagged parental and ZNF217 overexpressing OVCA420 cells into the intrabursal space around the ovary. ZNF217 overexpressing OVCA420 cells formed metastatic tumors on ovary and the peritoneal cavity whereas no tumor was observed in mice injected with control cells (Fig. 5M, S5G, S5H and S5I). Our in vivo data collectively establishes that ZNF217 is a powerful oncogene that can promote tumor progression and metastasis in a dose-dependent manner if its levels are elevated at either early or later stages in the metastatic process.

### ZNF217 increases cell proliferation and metastatic potential in immortalized fallopian tube epithelial cells

**Fig. 6:**
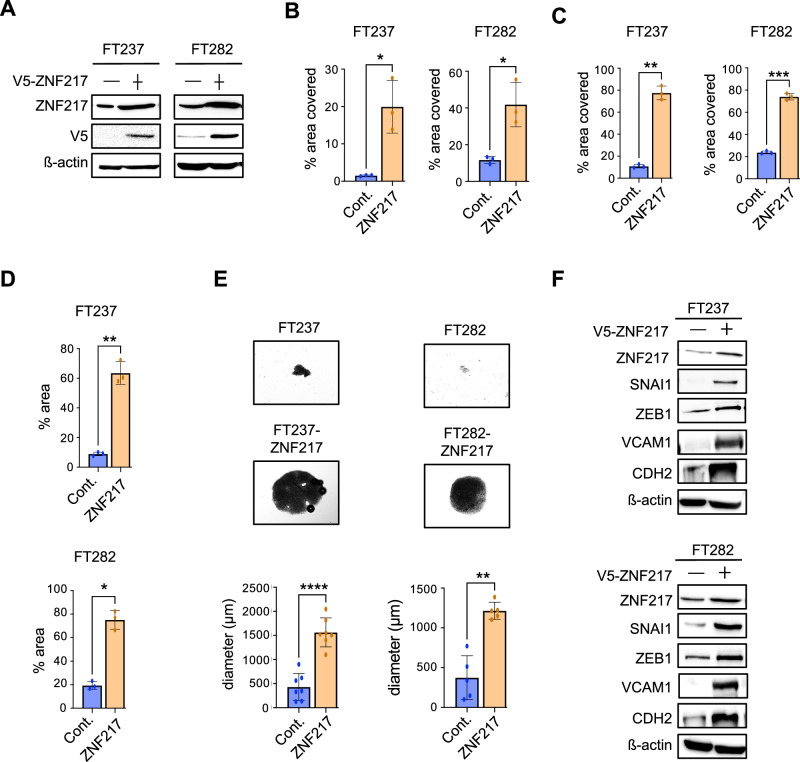
ZNF217 overexpression causes oncogenic transformation in immortalized fallopian tube secretory epithelial cells. **A** Western blot confirming stable ZNF217-V5 expression in FT237 and FT282 cells. **B** ZNF217 overexpression in FT237 and FT282 cells increases proliferation (n = 3). **C** Transwell migration assay shows that ZNF217 overexpression in FT237 and FT282 cells increases their ability to migrate (n = 3). **D** ZNF217 overexpression in FT237 and FT282 cells enhances their ability to invade through Matrigel (n = 3). **E** Representative bright field image and quantification showing that ZNF217 overexpression in FT237 (n = 7) and FT282 (n = 5) cells increases both the formation and size of spheroids when grown under low attachment conditions for 24 hours. **F** Western blot showing ZNF217 overexpression induces the expression of EMT associated genes in FT237 and FT282 cells.

HGSOCs have been shown to originate from the secretory epithelial cells of the fallopian tube^27–31^. To determine how ZNF217 impacts the early stages of HGSOC, we stably expressed V5-tagged ZNF217 in immortalized human fallopian tube epithelial cell lines, FT237 and FT282 (Fig. 6A). Like in ovarian cancer cells, ZNF217 overexpression caused a significant increase in cell proliferation (Fig. 6B and S6A), migration (Fig. 6C and S6B), and invasion through matrigel (Fig. 6D and S6C) in both FT237 and FT282 cells. Next, we investigated ZNF217’s effect on the ability of fallopian tube epithelial cells to survive anoikis. Detachment from the fimbrial basement membrane and the subsequent formation of spheroids are critical steps early in the successful dissemination of cancer cells from the primary tumor to secondary sites via the peritoneal cavity. Interestingly, ZNF217 overexpressing FT237 and FT282 cells formed several large spheroids when grown in suspension (Fig. 6E). In contrast, the control FT237 and FT282 cells were limited in their ability to aggregate effectively to form spheroids (Fig. 6E). Consistent with ZNF217’s ability to increase the metastatic potential of the fallopian tube epithelial cells, ZNF217 induced the expression of several EMT markers such as SNAI1, Zeb1, CDH2, and VCAM1 (Fig. 6F). These results suggest that ZNF217 overexpression at early stages of ovarian carcinogenesis can promote EMT which in turn could facilitate their transition into aggressive metastatic disease.

### ZNF217 promotes resistance to chemotherapeutic agents

**Fig. 7:**
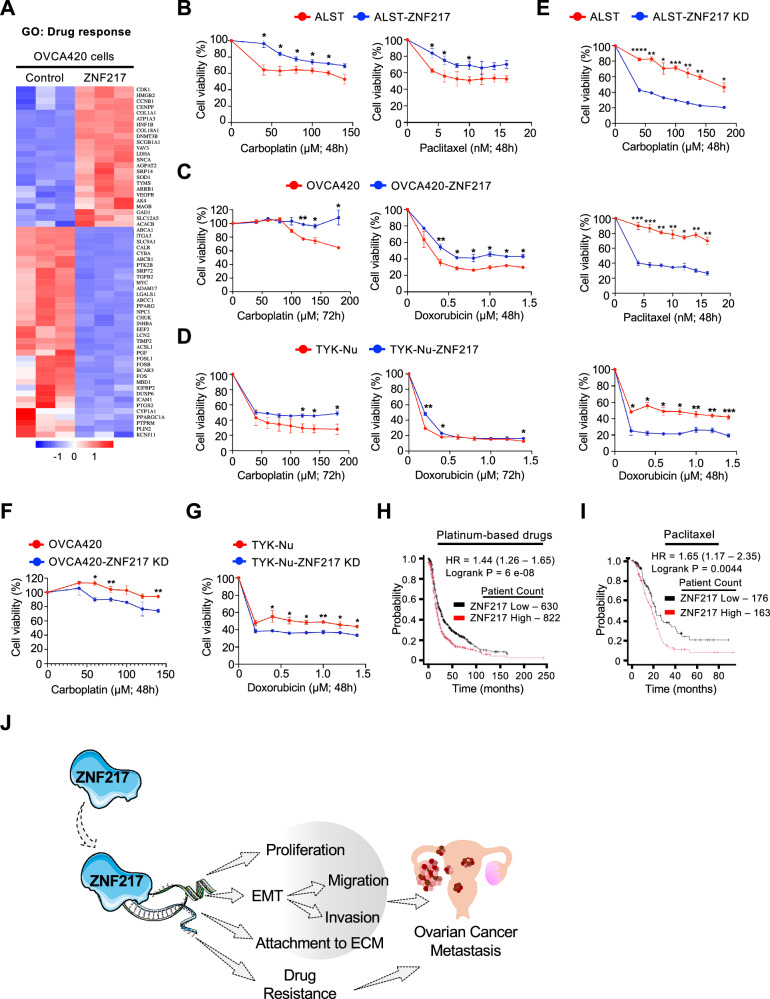
ZNF217 promotes chemotherapeutic resistance in ovarian cancer cells. **A** Gene ontology analysis from bulk RNAseq of OVCA420 cells revealed deregulation of genes implicated in drug response in ZNF217 overexpressing cells. Heatmap showing the differentially regulated genes that are related to drug response. **B** WST-1 cell viability assay shows that ZNF217 overexpression enhances the ability of ALST cells to resist carboplatin and paclitaxel treatment (n = 3). **C** WST-1 cell viability assay shows that ZNF217 overexpression increases the ability of OVCA420 cells to resist carboplatin and doxorubicin treatment (n = 3). **D** WST-1 cell viability assay shows that ZNF217 overexpression increases the ability of TYK-Nu cells to resist carboplatin and doxorubicin treatment (n = 3). **E** ZNF217 knockdown sensitizes ALST cells to carboplatin, doxorubicin and paclitaxel. n = 3. **F** ZNF217 knockdown sensitizes OVCA420 cells to carboplatin (n = 3). **G** ZNF217 knockdown sensitizes TYK-Nu cells to doxorubicin (n = 3). **H** Data retrieved from Kmplotter showing that ovarian cancer patients with high ZNF217 levels in their tumor respond poorly to chemotherapy containing platin. **I** Data retrieved from Kmplotter showing that ovarian cancer patients with high ZNF217 levels in their tumor respond poorly to chemotherapy containing paclitaxel. **J** Graphical summary of key findings of this study.

RNAseq analysis revealed that ZNF217 impacts cellular processes related to drug response (Fig. 7A). To determine how ZNF217 impacts chemoresistance, we treated control and ZNF217-overexpressing ovarian cancer cells to carboplatin, paclitaxel, and doxorubicin and measured their impact on cell viability. ZNF217 increased the ability of ALST cells to resist both carboplatin and paclitaxel (Fig. 7B and S7A). Greater resistance to carboplatin and doxorubicin was observed in both OVCA420 and TYK-Nu cells upon ZNF217 overexpression (Fig. 7C, D, and S7A). These data suggest that elevated ZNF217 in ovarian cancer cells promotes resistance to extant chemotherapeutics. Next, we tested if ZNF217 depletion would sensitize ovarian cancer cells to these chemotherapeutic agents. ZNF217 knockdown in ALST cells increased their sensitivity to carboplatin, doxorubicin, and paclitaxel (Fig. 7E, and S7B). Increased sensitivity to carboplatin and doxorubicin was also observed in OVCA420 and TYK-Nu cells respectively upon ZNF217 depletion (Fig. 7F, G, and S7B). To determine if there is a link between ZNF217 levels and chemoresistance in ovarian cancer patients we analyzed clinical data that are publicly available through the KM-plotter database. Consistent with our in vitro data, patients with ovarian tumors that express higher ZNF217 levels were less responsive to chemotherapy containing platinum-based drugs and paclitaxel (Fig. 7H, I). Collectively, our data show that ZNF217 is a potent oncogene that can impact ovarian cancer progression and metastasis by impacting multiple steps involved in this process and can also enable the tumor cells resist chemotherapeutics. These results also suggest that targeting ZNF217 will potentially slow down ovarian cancer metastasis and sensitize these tumors to extant chemotherapeutics resulting in increased patient survival.

## Discussion

Developing effective therapeutic strategies for metastatic ovarian cancer is contingent upon understanding factors that guide disease progression and drug resistance. Although ZNF217 is frequently overexpressed in ovarian cancer, how elevated ZNF217 impacts the different stages of ovarian carcinogenesis remain poorly understood^10^. A previous study suggested that ZNF217 could potentially function as an oncogene in ovarian cancer cells^32^. However, this study relied on data obtained using the HO-8910 cell line, which has been plagued with HeLa cell contamination and is designated to be a problematic cell line^33^. Further, the previous report did not investigate the effect of ZNF217 depletion in cancer cells nor address ZNF217’s effect on immortalized primary cells and therapeutic response in cancer cells. To address these limitations, we used multiple clinically relevant reagents and ovarian cancer models to comprehensively investigate how ZNF217 levels impacts the different steps in ovarian cancer metastasis and therapeutic response. Our data shows that ZNF217 promotes multiple aspects of tumor progression and metastasis, such as, cell proliferation and migration, ability to reorganize ECM, attach to ECM proteins and invade, resist anoikis to form multicellular aggregates, and chemo-resistance (Fig. 7J). Thus, by impacting several processes that are vital in the progression of primary tumor to lethal metastatic disease, ZNF217 acts as a potent oncogene in ovarian cancer cells. Our data also shows that ZNF217 can impact ovarian cancer progression at several different stages including the transition of early-stage tumors to aggressive metastatic disease. ZNF217 overexpression in immortalized fallopian tube cells increased proliferation, migration, and invasive potential in these cells (Fig. 6). We observed that ZNF217 induces the expression of molecular markers associated with EMT in these cells. However, it is still unknown if ZNF217 levels are elevated in early stages of ovarian cancer in patients such as in the pre-cancerous serous tubal intraepithelial carcinoma (STIC) lesions. Future studies focusing on ZNF217 levels in these pre-cancerous lesions will help us better understand ZNF217’s role and its clinical significance during the early stages of ovarian cancer progression.

As mutations in p53 are extremely frequent in ovarian cancer and can impact disease progression and therapeutic response, it is critical to determine if the p53-status of the tumor cells influences the observed oncogenic effects of ZNF217 overexpression^19,20,34^. ZNF217 promoted oncogenic phenotypes in all ovarian cancer cells we tested so far, demonstrating that ZNF217 functions as an oncogene independent of the p53 mutation status of cancer cells and can promote tumor progression and therapeutic resistance in a broad spectrum of ovarian cancer patients who differ in their tumor p53 status. Although transcription factors are known to impact cellular functions by altering gene expression programs, they can also exert transcription-independent effects through specific protein-protein interactions. To ascertain if ZNF217’s role as an oncogene in ovarian cancer cells is dependent on its ability to function as a transcription factor, we tested if the ZNF217 DNA binding mutant (ZNF217-H489A) can rescue the phenotypes observed upon ZNF217 knockdown. Unlike wild-type ZNF217, the ZNF217-H489A mutant was unable to rescue pro-metastatic phenotypes suggesting that ZNF217’s transcriptional function is critical for promoting metastasis. It is important to note that these results do not suggest that the transcription-independent protein-protein interactions mediated by ZNF217 are not important for its oncogenic function. Previous data have characterized ZNF217’s role in protein complexes with HDACs and Co-REST in promoting a repressive chromatin state^23,35^. However, our findings suggest that ZNF217’s activity as a transcriptional activator is equally important in driving pro-metastatic gene expression. We compared the differentially expressed genes identified in ZNF217 overexpressing ovarian cancer cell lines to a previously published dataset of direct ZNF217 targets in the breast cancer cell line MCF7 and identified that there was significant overlap between them (Fig. 4C)^26^. However, we acknowledge that ChIP-seq data from MCF7 cells may not fully represent the context of ovarian cancer cells. Therefore, future studies aimed at identifying direct downstream targets of ZNF217 in ovarian cancer cells will help further understand how ZNF217’s mediates its oncogenic effects in these cells.

Interestingly, our data reveal that ZNF217 overexpression also enables ovarian cancer cells to resist extant chemotherapeutics and can thus contribute to the poor clinical outcome (Fig. 7J). ZNF217 depletion impairs ovarian cancer metastases and sensitizes them to extant chemotherapeutics such as carboplatin, paclitaxel, and doxorubicin. These data highlight the untapped potential of ZNF217 as a therapeutic target in metastatic ovarian cancer. Therapeutically targeting a transcription factor such as ZNF217 will need either a PROTAC-based strategy or knowledge of druggable upstream regulators. As ZNF217 is overexpressed in several cancers (Fig. S1A), these approaches will likely benefit patients well beyond ovarian cancer.

## Methods

### Cell lines, culture conditions, and reagents

Human ovarian cancer cells were cultured in RPMI medium (Thermo Fisher, #11875119) supplemented with 10% FBS (Omega Scientific, #13083) and 1% penicillin and streptomycin (VWR, #16777-164). FT282 and FT237 were cultured in DMEMF12/Hamm’s 50:50 (VWR, #45000-344) supplemented with 10% FBS and 1% penicillin and streptomycin. HEK293T cells were cultured in DMEM media supplemented with 10% FBS and 1% penicillin and streptomycin. All cells were grown at 37 °C with 5% CO_2_.

### Lentiviral transduction

9 µg of lentiviral plasmid DNA prep was combined with 9 µg pSPAX2 (lentiviral packaging vector), 0.9 µg pMDG2.G (envelope vector), and 57 µl of attractene reagent (Qiagen, #301005) and brought up to 500 µl volume with Gibco Opti-MEM media (Fisher Scientific, #31-985-062) and incubated at room temperature for 30 minutes. Transfection mix was added dropwise to a 60–70% confluent 10 cm plate of HEK293T cells. Media containing lentivirus particles were collected at 24 h, 48 h, 72 h, and filtered with a 0.45 µm syringe filter. 1 ml of lentivirus containing media was used to transduce the recipient ovarian cancer cells. Cells were selected using 5–10 µg/ml of puromycin for ZNF217-V5 constructs, 100 µg/ml of G418 for lenti-luciferase virus, or 80–100 µg/ml of hygromycin for the PLVX-IRES-ZNF217-WT or ZNF217-H489A rescue vector constructs. OVCA420 and ALST cells expressing ZNF217-RFP and their corresponding empty vector FUCRW controls were selected by fluorescence activated cell sorting (FACS) using RFP intensity as a surrogate for ZNF217 levels. During FACS, these cells were gated for high- and low-RFP (ZNF217) expressing populations to obtain ZNF217-high and ZNF217-low populations. ZNF217 expression in these cells were confirmed by Western Blot. Cell lines stably transfected with the corresponding empty vector were used as controls in all our studies. For experiments using OVCA420 and ALST cell lines, parental OVCA420 and ALST cells transfected with empty FUCRW vector were used as controls. Similarly, for experiments using TYK-Nu, OVCAR3, OVCAR4, and OVCAR8 cell lines, the respective cell line stably transfected with empty pLVX-puro vector were used as control. All shRNA constructs were purchased from Sigma-Aldrich (mission lentiviral shRNA). pLKO.1 puro-shZNF217-1 was used to deplete ZNF217 in OVCA420 and OVCAR4 cells. pLKO.1 puro-shZNF217-3 was used to deplete ZNF217 in TYK-Nu cells. pLKO.1 puro-shZNF217-4 was used to deplete ZNF217 in OVCAR3 cells and pLKO.1 puro-shZNF217-5 was used to knockdown ZNF217 in OVCAR8 cells. Please see Table S5 for information on the shRNA sequences used in this study.

### Western blot

Cells were lysed using either RIPA buffer containing protease and phosphatase inhibitors or using a lysis buffer containing 8 M urea, 50 mM NaH_2_PO_4_ and 300 mM NaCl. Protein concentration was determined using Bio-Rad protein assay reagent. Equal amounts of total protein were run on 10% SDS PAGE gel and transferred to a 0.45 µm PVDF membrane. The transferred membrane was blocked for 1 hour at room temperature in 3% milk. Primary antibodies were added overnight at 4 °C. Membranes were washed 4 times in 10 ml of 1X TBST. Secondary antibody is incubated on a shaking incubator for 1 h at room temperature. Membranes are washed 4 times in 1X TBST and developed using ECL solution and a BioRad ChemiDoc Imager. Please see Table S6 for information on the list antibodies used for western blot in this study.

### Proliferation assay

10,000 cells were plated in a 6-well plate and allowed to grow for 3–7 days (depending on the cell line). Cells were fixed in 4% paraformaldehyde, washed with 1x PBS, stained with 0.5% crystal violet, and imaged using a 4X objective. Cell count and percent area of cells is calculated using ImageJ from at least 3 different biological replicates (4 fields/sample). For counting cells, the threshold was adjusted to black and white, and measurements were set to count individual cells detected by the threshold adjustment. Cell counts were acquired by analyzing particles with numerical overlay to confirm the accurate capture of all cells.

### WST-1 Proliferation assay

5000 cells were counted and seeded into a 96 well plate in triplicate and allowed to grow at 37 °C in CO_2_ incubator. WST-1 assay was performed 24 h and 48 h later Number of viable cells were determine using WST-1 reagent (Sigma Aldrich, #11 644 807 001) at 24 and 48 hours as per manufacturer’s instructions. Colorimetric reading was acquired using BioTek Gen5 plate reader.

### Wound healing assay

Cells (7 × 10^5^ cells/ml) were seeded into an IBIDI cell culture insert on a 12-well plate and allowed to grow for 24 h at 37 °C. At 24 h, the cell culture insert was removed, and images were captured at 4 h intervals. Percent area of cells was calculated for an overall change in area to assess the migratory ability of cells using an ImageJ plugin developed for migration assay analysis^36^.

### Transwell migration assay

10^5^ cells in 0.5 mL 10% RPMI was plated inside 12-well permeable inserts. 1 mL RPMI containing 20% FBS was added to the 12-well dish, beneath the permeable insert. 24 h later the cells were fixed in 4% PFA and stained using 0.5% crystal violet. Excess crystal violet was washed, the permeable insert was removed, mounted on a microscope slide, and imaged at 4X magnification. Percentage area was calculated using ImageJ.

### Matrigel invasion assay

12-well permeable inserts were coated with 40 µl of a 1:3 ratio of 1 mg/ml Matrigel (Corning) to serum-free RPMI medium. Matrigel is allowed to set for 1 h at room temperature. 10^5^ cells were reconstituted in 500 µl of RPMI media with 10% FBS. 1 mL of RPMI media with 20% FBS is added to the bottom of the 12-well dish, beneath the permeable insert. The assay was carried out for 24 h at 37 °C, following which the cells were rinsed with PBS, fixed for 15 minutes using 4% PFA, washed again with PBS and stained for 10 minutes using 0.5% crystal violet. Excess crystal violet was rinsed, and the permeable insert was then removed from the well insert and mounted on a microscope slide and imaged at 4X magnification. Percentage area was calculated in ImageJ.

### Electric Cell-substrate Impedance Sensing (ECIS) assay

OVCA420 and OVCA420-ZNF217 were seeded in ECIS chambers at different densities (15,000/20,000/30,000/40,000 cells per well) in 200 µL media. A total of 3 ECIS chambers were used for each cell type. 2 days after the cells were seeded, media was replaced, and chambers were moved to the ECIS machine for measurement. The resistance/capacitance and conductance for the cells were measured at multiple frequencies (62.5 Hz/125 Hz/250 Hz/500 Hz/1 kHz/2 kHz/4 kHz/8 kHz/16 kHz/32 kHz/64 kHz). The measurements were made every 3 min for a duration of 48 h.

### Adhesion assay

Recombinant human collagen-1a or human plasma fibronectin (5 µg/ml in PBS) were coated on 96-well plate. Cells were added to the well and incubated at 37 °C for 1 h. 1 × 10^4^ cells were used for ZNF217 overexpression studies, 5 × 10^4^ cells were used for ZNF217 knockdown studies. Cells were fixed in 4% PFA, washed with PBS and stained using 0.2% crystal violet. Adherent cells were counted using a cell counter plugin (https://imagej.net/ij/plugins/cell-counter.html).

### Spheroid assay

The hanging drop method was performed as described previosuly^37^. 2 × 10^6^ cells in 1 mL RPMI were suspended on the lid of a 6-well plate, with 3 mL of PBS in the dish. After 24 h, cells were moved to an untreated round bottom 96-well plate and placed in a shaking incubator at 37 °C until spheroids form.

### RT-qPCR analysis

Total RNA was extracted from cells and cDNA was prepared using ZymoScript one-step RT-qPCR kit (Cat#R3014). Quantitative PCR (qPCR) was performed using SYBR Green qPCR Master Mix (K0221, Thermo Scientific) on a CFX Real-Time PCR Detection System (Bio-Rad). Cycling conditions was 95 °C for 10 minutes, 40 cycles of 95°C for 15 seconds, 72 °C for 15 seconds, followed by melt curve analysis. qPCR amplifications were performed in technical triplicates as well as biological triplicates. The EMT primer array was purchased from Real Time Primers (#HEMT-I). Please see Key Table S7 for information on the list of RT-qPCR primers used in this study.

### RNA sequencing and data analysis

Flash frozen cell pellets were provided to Azenta Life Sciences who performed RNA extraction and RNA sequencing using their standardized procedure. Three biological replicates were submitted for each sample. Proper quality check was included to ensure data quality. Preliminary data analysis was performed by Azenta Life Sciences as part of the package. Genes with an adjusted p-value < 0.05 and absolute log2 fold change >1 was considered significant. Volcano plot and heatmap were generated using the SRPlot online platform^38^.

### Molecular cloning

ZNF217 was amplified by PCR using primers that allows their restriction digestion and ligation into the desired vector. PCR products were gel-purified (Zymoclean gel DNA recovery kit, catalog #D4001) and ligated with the desired vector that was digested with the respective enzymes. Ligation was performed using T4 DNA Ligase (NEB, #M0202L) either for 1 hour or overnight at room temperature. Ligation product was transformed into chemically competent DH5-alpha cells, plated onto LB-Agar-Ampicillin plates and incubated overnight at 37 °C. Colonies were screened with colony PCR and confirmed by Sanger sequencing. ZNF217_pLX307 was a gift from William Hahn and Sefi Rosenbluh (RRID:Addgene_98384)^28^. Please see Table S8 for information on the list of primers used for cloning in this study.

### Animal experiments and in vivo tumor imaging

Two strains of immune compromised mouse strains, Foxn1 nude and NSG, were used in this study. Animals were housed 2–5 per cage in temperature (22 ± 2 °C) and humidity (55% ± 15%) controlled rooms on a 12-hour light/dark cycle. Water and food were available to animals ad libitum. Animals are monitored daily for tumor growth and sacrificed when they meet euthanasia criteria outlined by NIH’s Guide for the Care and Use of Laboratory Animals. For intraperitoneal model, 10^7^ cells in 500 µL media was injected into the lower left quadrant of the intraperitoneal cavity of female mice. For intrabursal model, 50,000 cells in 5 µL PBS was injected into the exposed ovary bursa of 10-week-old Foxn1 nude mice as described previously^39^. The IVIS system (Perkin Elmer) was used for in vivo luciferase quantification to measure tumor growth. Intrabursal injection and in vivo imaging was carried out under anesthesia, which was achieved using isoflurane (3–4% for induction and 1-2% for maintenance). Signal intensity was quantified by Perkin Elmer’s Living Image analysis software. All animal studies were performed under a protocol approved by the Institutional Animal Care and Use Committee at the University of Maryland, Baltimore County. As ovarian cancer is a female-specific disease, we used only female age-matched mice were used.

### Quantification and statistical analysis

All in vitro experiments were performed at least three times (three biological replicates with three technical replicates each). Data is presented as mean ± standard deviation with individual data points unless otherwise noted. Statistical significance was measured using student’s two-tailed t-test. The following were considered significant: p < 0.05 (*), p < 0.005 (**), p < 0.001 (***), p < 0.0001 (****). RNAseq was performed in biological triplicates. Distribution of read counts in libraries were examined before and after normalization. The original read counts were normalized to adjust for various factors such as variations of sequencing yield between samples. These normalized read counts were used to accurately determine differentially expressed genes. The overall similarity among samples were assessed by the euclidean distance between samples. This method was used to examine which samples are similar/different to each other and if they fit to the expectation from the experiment design. A comparison of gene expression between the groups of samples was performed using DESeq2. The Wald test was used to generate p-values and log2 fold changes. Genes with an adjusted p-value < 0.05 and absolute log2 fold change > 1 were called as differentially expressed genes. Significantly differentially expressed genes were clustered by their gene ontology and the enrichment of gene ontology terms was tested using Fisher exact test (GeneSCF v1.1-p2). Survival studies in mice were analyzed by Kaplan-Meier curves and log-rank (Mantel-Cox) tests.

## Supplementary information

Supplemental data_Wardrup K et al.

Table S1.

Table S2.

Table S3.

Table S4.

ARRIVE Compliance Questionnaire.

## Data Availability

All data supporting the findings of this study are available within the article and its supplementary file. RNAseq data used in the paper are available in the Sequence Read Archive (SRA) database under accession code PRJNA1353133. There are no restrictions on data availability.
