# Supplementary material for: ZNF217 promotes ovarian cancer progression by impacting multiple pivotal steps in the metastatic process
Source: NPJ Precis Oncol. 2025 Dec 4;9:392. doi: 10.1038/s41698-025-01153-8 (PMC12678582; doi:10.1038/s41698-025-01153-8)
Supplement: Supplementary file 1 — Supplemental data_Wardrup K et al. [file 41698_2025_1153_MOESM1_ESM.pdf]

SUPPLEMENTAL FIGURES AND FIGURE LEGENDS

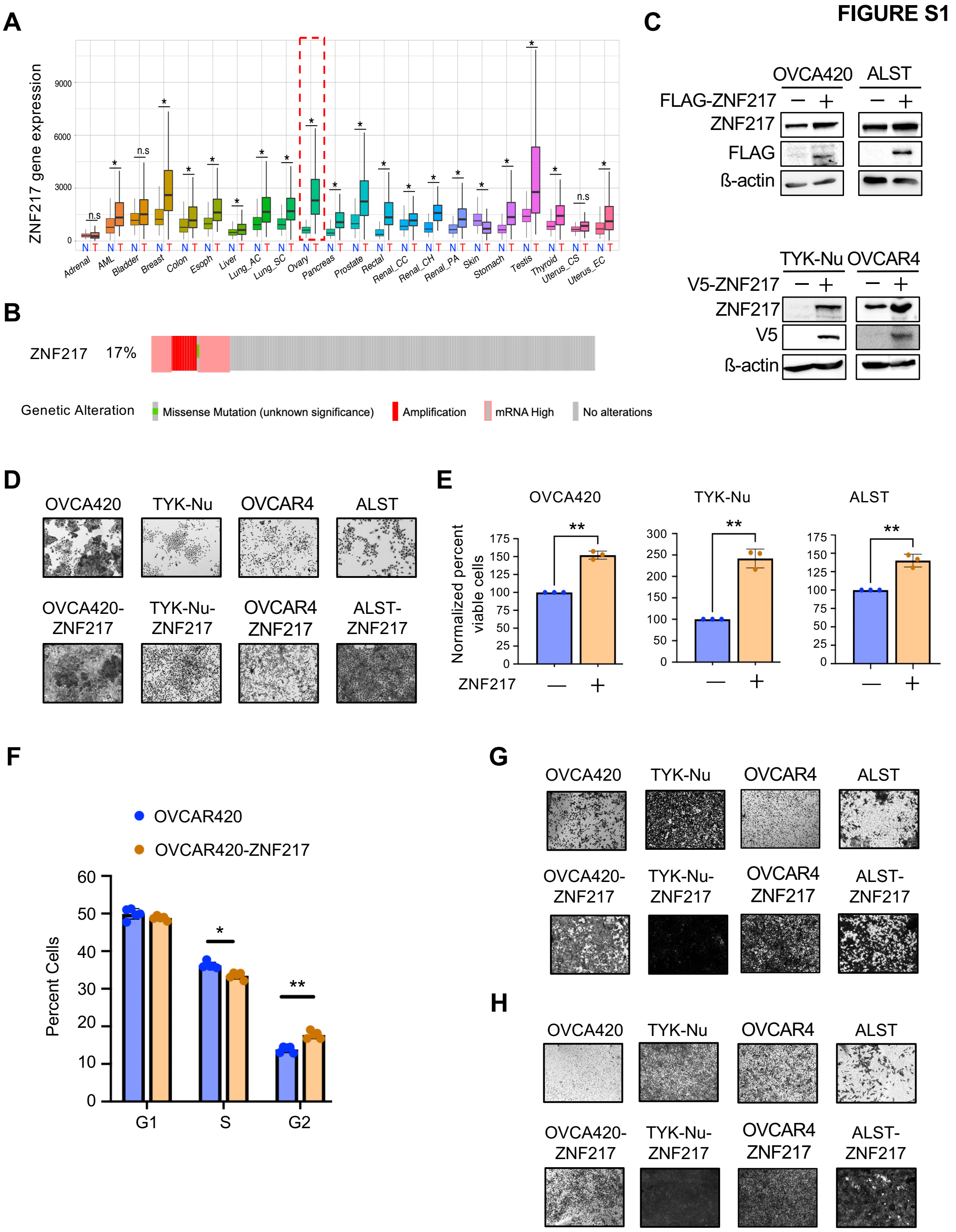

FIGURE S1 (cont.)

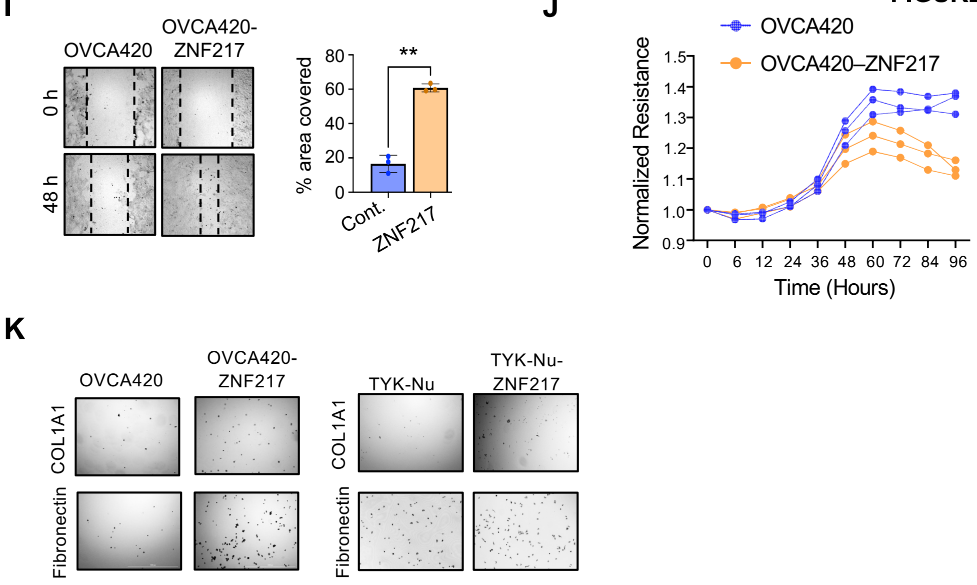

Figure S1. ZNF217 overexpression drives proliferation and metastatic phenotypes in ovarian cancer cells in vitro.

A. ZNF217 mRNA is elevated in multiple cancers. Data retrieved from TNMplot.

B. Data from The Cancer Genome Atlas Database (Firehose Legacy dataset) show that ZNF217 is amplified or overexpressed in 17 percent ovarian cancer cases.

C. Western blot confirming stable ZNF217 overexpression in OVCA420, ALST, TYK-Nu, and OVCAR4 cells.

D. Representative bright field images showing the effect of stable ZNF217 overexpression on cell proliferation in ovarian cancer cells.

E. WST-1 cell proliferation assay showing ZNF217 overexpression increases proliferation in ovarian cancer cells (n=3).

F. Cell cycle analysis using cells in sub-confluent culture showing differences in cell cycle distribution between OVCA420 and OVCA420-ZNF217 cells (n=5).

G. Representative bright field images showing the effect of stable ZNF217 overexpression on cell migration (transwell migration assay) in ovarian cancer cells.

H. Representative bright field images showing the effect of stable ZNF217 overexpression on the ability of ovarian cancer cells to invade through Matrigel (Matrigel invasion assay).

I. Bright field representative images and quantification (n=3) at 0 and 48 hours from a wound healing assay showing that ZNF217 increases cell migration in OVCA420 cells that are treated with proliferation inhibitor, mitomycin C (MMC).

J. Individual runs from ECIS assay that was used to plot Fig. 2G.

K. Representative bright field images of control and ZNF217 overexpressing (stable overexpression) OVCA420 and TYK-Nu cells on tissue culture plates coated with fibronectin and COL1A1. The cells were allowed to attach to these plates for 45 min before taking these images.

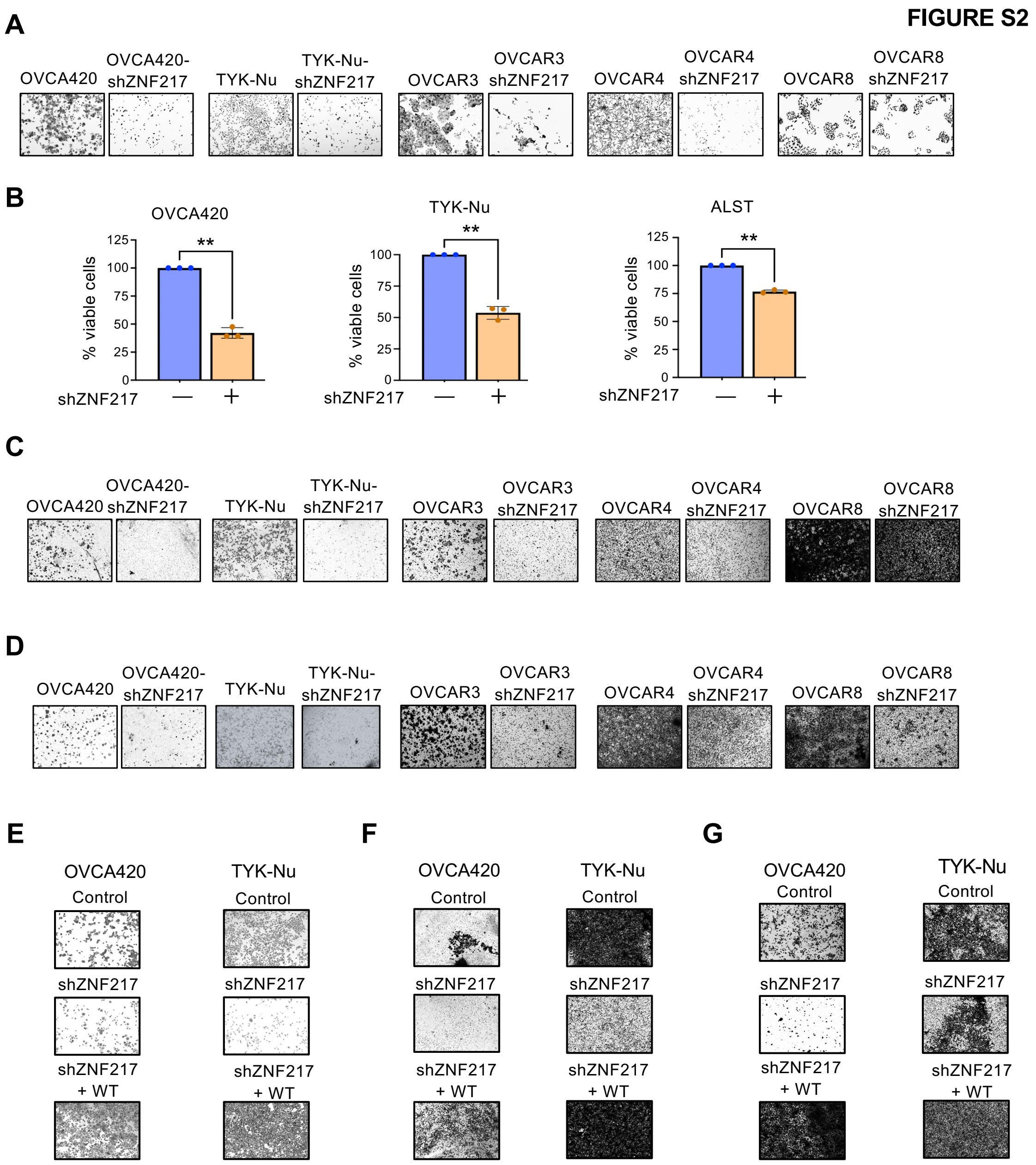

Figure S2. ZNF217 depletion impairs ovarian cancer cell proliferation and metastatic potential in vitro.

A. Representative bright field images showing the effect of stable ZNF217 knockdown on cell proliferation in ovarian cancer cells.

B. WST-1 cell proliferation assay showing ZNF217 knockdown decreases proliferation in ovarian cancer cells (n=3).

C. Representative bright field images showing the effect of stable ZNF217 knockdown on cell migration (transwell migration assay) in ovarian cancer cells.

D. Representative bright field images showing the effect of stable ZNF217 knockdown on the ability of ovarian cancer cells to invade through Matrigel (Matrigel invasion assay).

E. Representative bright field images showing the effect of rescuing ZNF217 expression using shRNA-resistant ZNF217 in ZNF217 knockdown ovarian cancer cells on cell proliferation.

F. Representative bright field images showing the effect of rescuing ZNF217 expression using shRNA-resistant ZNF217 in ZNF217 knockdown ovarian cancer cells on cell migration (transwell migration assay).

G. Representative bright field images showing the effect of rescuing ZNF217 expression using shRNA-resistant ZNF217 in ZNF217 knockdown ovarian cancer cells on invasion (Matrigel invasion assay).

FIGURE S3

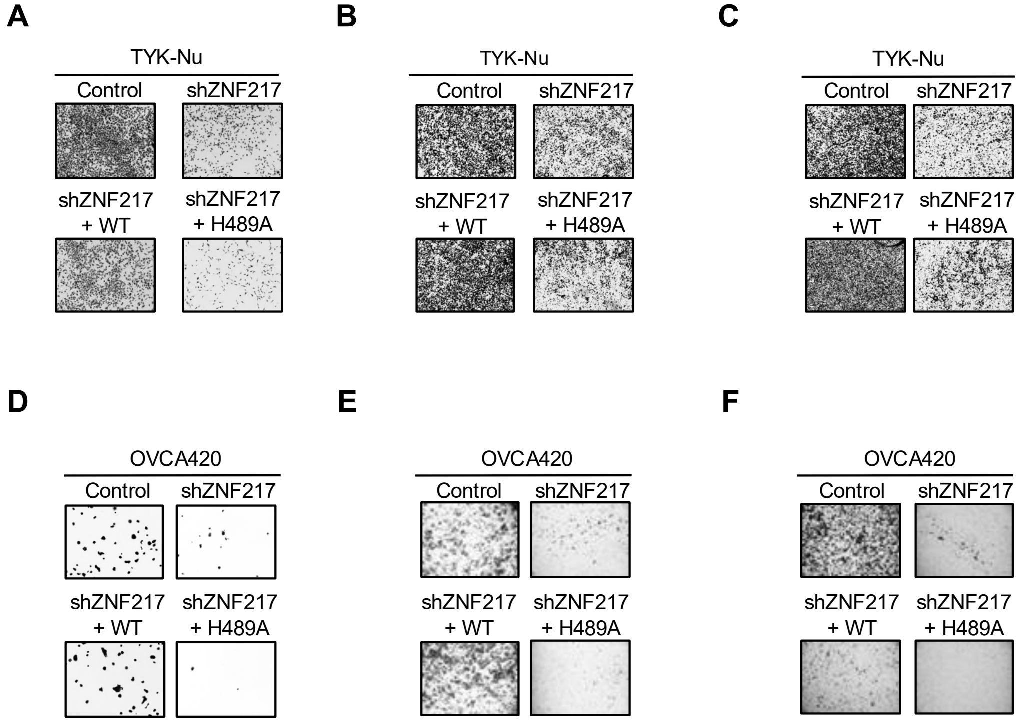

Figure S3. Oncogenic activity of ZNF217 in ovarian cancer cells is dependent on its ability to bind DNA.

A. Representative bright field images showing that proliferation defects due to ZNF217 depletion in TYK-Nu cells can be rescued by an shRNA-resistant ZNF217-WT expression, but not ZNF217-H489A.

B. Representative bright field images of transwell migration assay showing that ectopic expression of an shRNA-resistant ZNF217-WT, but not ZNF217-H489A, rescues defects in TYK-Nu migration induced upon ZNF217 knockdown.

C. Representative bright field images showing that unlike ZNF217-WT, ZNF217-H489A mutant does not rescue the impact of ZNF217 depletion on the ability of TYK-Nu cells to invade through the Matrigel.

D. Representative bright field images showing that proliferation defects due to ZNF217 depletion in OVCA420 cells can be rescued by an shRNA-resistant ZNF217-WT expression, but not ZNF217-H489A.

E. Representative bright field images of transwell migration assay showing that ectopic expression of an shRNA-resistant ZNF217-WT, but not ZNF217-H489A, rescues defects in OVCA420 migration induced upon ZNF217 knockdown.

F. Representative bright field images showing that unlike ZNF217-WT, ZNF217-H489A mutant does not rescue the impact of ZNF217 depletion on the ability of OVCA420 cells to invade through the Matrigel.

FIGURE S4

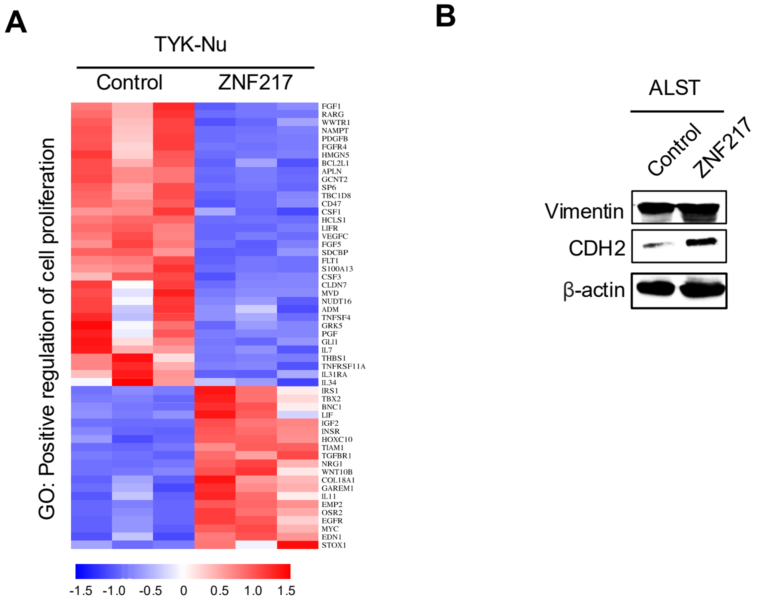

Figure S4. ZNF217 overexpression impacts transcription of genes important in driving ovarian cancer cell proliferation and EMT.

A. Heatmap showing differentially regulated genes in the gene ontology terms positive regulation of cell proliferation.

B. Western blot showing the stable ZNF217 overexpression causes an increase in the levels of vimentin and N-cadherin (CDH2) in ALST cells. Both these proteins are associated with a more mesenchymal phenotype.

FIGURE S5

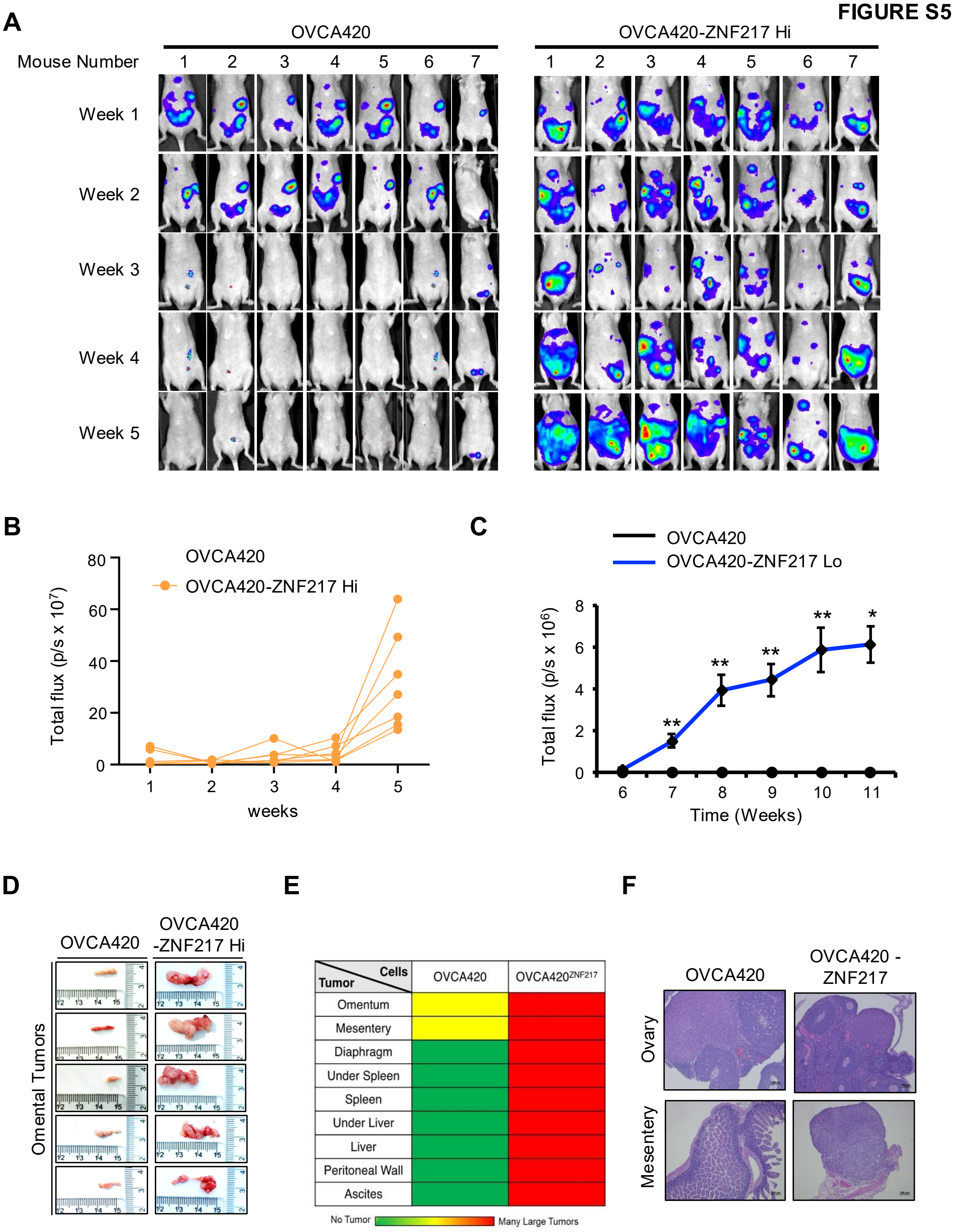
A
OVCA420
OVCA420-ZNF217 Hi
Mouse Number
1 2 3 4 5 6 7
Week 1
Week 2
Week 3
Week 4
Week 5
B
Total flux (p/s x 10^7)
OVCA420
OVCA420-ZNF217 Hi
weeks
C
OVCA420
OVCA420-ZNF217 Lo
Total flux (p/s x 10^6)
Time (Weeks)
D
OVCA420
OVCA420 -ZNF217 Hi
Omental Tumors
E
Cells
Tumor
OVCA420
OVCA420ZNF217
Omentum
Mesentery
Diaphragm
Under Spleen
Spleen
Under Liver
Liver
Peritoneal Wall
Ascites
No Tumor
Many Large Tumors
F
OVCA420
OVCA420 - ZNF217
Ovary
Mesentery

FIGURE S5 (cont.)

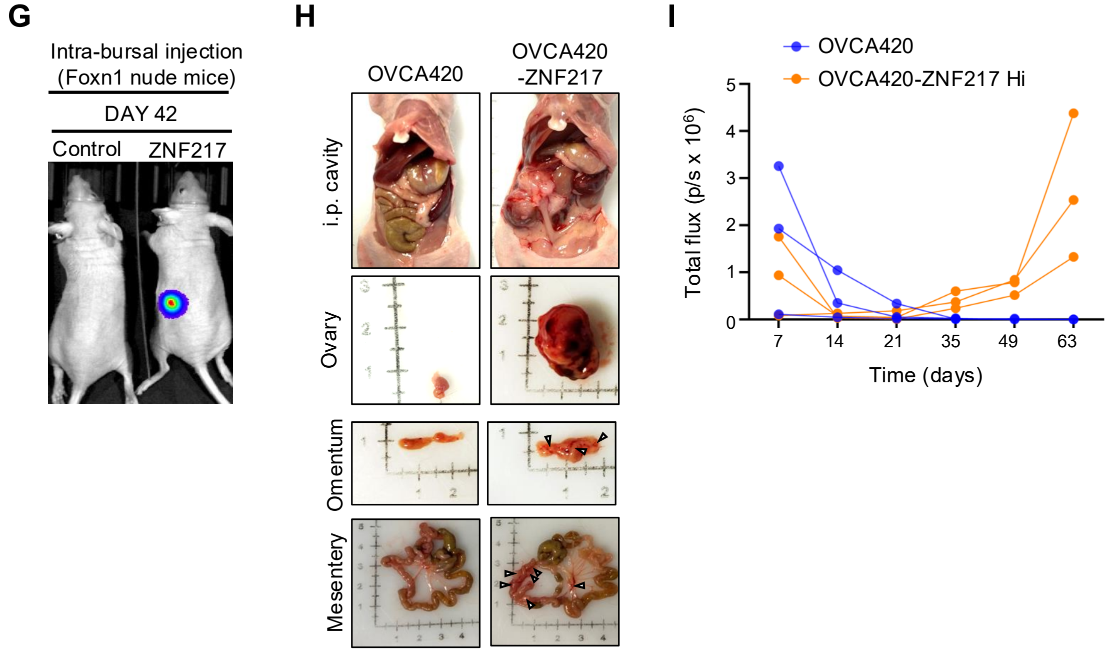

Figure S5. ZNF217 overexpression promotes ovarian cancer metastasis in vivo.

A. Non-invasive luciferase imaging using IVIS shows increased tumor burden in female foxn1 nude mice i.p injected with OVCA420-ZNF217 Hi cells compared control OVCA420 cells.

B. Quantification of luciferase intensity over time for individual mice i.p injected with OVCA420 and OVCA40-ZNF217 Hi cells. Cumulative representation of this data is shown in Figure 6C.

C. Quantification of luciferase intensity acquired through in vivo imaging over time reveals that in Foxn1 nude mice i.p injected with control and OVCA420-ZNF217 Lo cells, ZNF217 expression results in significantly higher metastatic tumor burden over time (n=6). The kinetics of progression is slower in OVCA420-ZNF217 Lo tumors compared to OVCA420-ZNF217 Hi tumors (see Figure 6C).

D. Images of metastatic tumors on omentum in Foxn1 nude mice i.p injected with OVCA420 and OVCA420-ZNF217 Hi cells.

E. The tumor burden in common ovarian cancer metastatic sites in Foxn1 nude mice i.p injected with OVCA420 and OVCA420-ZNF217 Hi cells.

F. Hematoxylin and eosin (H&E) stain of OVCA420 control and ZNF217-high expressing tumors reveal minimal tumor burden in control. By contrast large metastatic lesions are observed in ZNF217 expressing cohorts. H&E for tumors on ovary and mesentery are shown.

G. Representative in vivo imaging of luciferase intensity on day 42 in female Foxn1 nude mice injected intrabursally with OVCA420 control and OVCA420-ZNF217-Hi cells. Intrabursal injection of OVCA420-ZNF217-Hi cells results in larger tumor burden.

H. ZNF217 expression in OVCA420 cells increases tumor forming ability in the peritoneal cavity, ovary, omentum, and mesentery in an ovarian intrabursal injection model using female immunodeficient Foxn1 nude mice.

I. Quantification of luciferase intensity over time for individual mice i.b injected with OVCA420 and OVCA40-ZNF217 Hi cells. Cumulative representation of this data is shown in Fig. 6M.

FIGURE S6

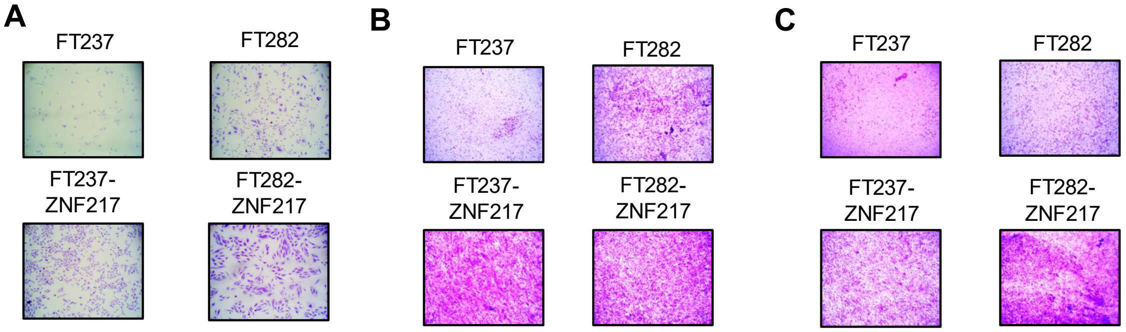

Figure S6. ZNF217 overexpression causes oncogenic transformation in immortalized fallopian tube secretory epithelial cells.

A. Representative bright field images showing the effect of stable ZNF217 overexpression on cell proliferation in FT237 and FT282 cells.

B. Representative bright field images showing the effect of stable ZNF217 overexpression on cell migration (transwell migration assay) in FT237 and FT282 cells.

C. Representative bright field images showing the effect of stable ZNF217 overexpression on the ability of FT237 and FT282 cells to invade through Matrigel (Matrigel invasion assay).

FIGURE S7

A

	ALST	ALST-ZNF217
Carboplatin (IC50; µM)	139.5	312.6
Paclitaxel (IC50; nM)	11.14	24.79

	OVCA420	OVCA420-ZNF217
Carboplatin (IC50; µM)	597.9	6.248e+45
Doxorubicin (IC50; µM)	0.33	0.66

	TYK-Nu	TYK-Nu-ZNF217
Carboplatin (IC50; µM)	42.55	82.03
Doxorubicin (IC50; µM)	0.111	0.164

B

	ALST	ALST- ZNF KD
Carboplatin (IC50; µM)	205.8	38.31
Paclitaxel (IC50; nM)	38.61	4.573
Doxorubicin (IC50; µM)	0.634	0.1443

	OVCA420	OVCA420-ZNF217 KD
Carboplatin (IC50; µM)	8.563e+56	524.9

	TYK-Nu	TYK-Nu-ZNF217 KD
Doxorubicin (IC50; µM)	0.673	0.351

Figure S7. ZNF217 promotes chemotherapeutic resistance in ovarian cancer cells.

A. IC50 values for carboplatin, paclitaxel, and doxorubicin in control and ZNF217 overexpressing ALST, OVCA420 and TYK-Nu cells (N=3).

B. IC50 values for carboplatin, paclitaxel, and doxorubicin in control and ZNF217 knockdown ALST, OVCA420 and TYK-Nu cells (N=3).

SUPPLEMENTARY TABLES

Table S1. List of differentially expressed genes in OVCA420 and OVCA420-ZNF217. See attached excel file names Table S1.

Table S2. List of differentially expressed genes in TYK-Nu and TYK-Nu-ZNF217. See attached excel file names Table S2.

Table S3. List of common genes that are differentially regulated in both OVCA420 and TYK-Nu cells upon ZNF217 overexpression. See attached excel file names Table S3.

Table S4. List of genes that are differentially regulated in OVCA420 and TYK-Nu cells that have been identified as direct ZNF217 transcriptional targets in MCF-7 cells. See attached excel file named Table S4.

**shRNA sequence**	**Source**	**Identifier**
pLKO.1 puro-shZNF217-1 - CCCAAATCTATTGTTGTCTTA	Sigma (Mission shRNA)	TRCN0000013058
pLKO.1 puro-shZNF217-2 - ACACATTCAGAAGACCTTAAT	Sigma (Mission shRNA)	TRCN0000369268
pLKO.1 puro-shZNF217-3 - TGTAAACCTGCTTGTTCAAAT	Sigma (Mission shRNA)	TRCN0000364635
pLKO.1 puro-shZNF217-4 - CCGGCAATTTCTTTGAGTAAA	Sigma (Mission shRNA)	TRCN0000013061
pLKO.1 puro-shZNF217-5 - GCCAACTCAATCCCTCTTAAT	Sigma (Mission shRNA)	TRCN0000013060

Table S5. ZNF217 shRNA sequence. ZNF217 shRNA was ordered from Sigma-Aldrich Mission shRNA. These sequences were purchased as a plasmid in the pLKO.1-puro plasmid.

Antibody	SOURCE	IDENTIFIER
ß-actin	Sigma	A2228
E-cadherin (CDH1)	Proteintech	20874
FLAG	Sigma	F1804
KRT7	Proteintech	22208
N-cadherin (CDH2)	Proteintech	22018
PAX8	Proteintech	10336
SNAI1	Proteintech	13099
TWIST1	Proteintech	25465
V5 tag	Proteintech	14440
VCAM1	Proteintech	11444
Vimentin	Proteintech	10366
ZEB1	Proteintech	21544
ZNF217	Thermo Fisher	720352

Table S6. List of antibodies used in this study.

Gene	Primer Sequence
ZNF217	Fwd: AAACATGCCAACTCAATCCCTC<br>Rev: GGAATGGAACAACAGCGGT
ß-actin	Fwd: GCTCTTTTCCAGCCTTCCTT<br>Rev: CGGATGTCAACGTCACACT
SNAI1	Fwd: TGCCCTCAAGATGCACATCCGA<br>Rev: GGGACAGGAGAAGGGCTTCTC

Table S7. List of RT-qPCR primers used in this study.

Construct	Primer	Sequence	NEB Enzyme
FU-CRW _FLAG-ZNF217	FLAG-ZNF217 Forward	GTACTCTAGAATGGACTACAAAGACGATGACGA	Xba1
	ZNF217 Reverse	CATGGAATTCTCAAGTTTTTTTGTCATTTGGTC	EcoR1
PLVX-IRES-BERKY3:FLAG-ZNF217	FLAG-ZNF217 Forward	TGGCACCGGTATGGACTACAAAGACGATGACGACAAGCAATCGAAAGTG	Age1
	ZNF217 Reverse	GCGGCCGCTCTATCAAGTTTTTTTGTC	Not1
PLVX-IRES-BERKY3:FLAG-ZNF217-H489A site directed mutagenesis	ZNF217-H489A Forward	CAAATTATTACCTCAATATTGCCCTCAGAACGCATACAGGTG	N/A
	ZNF217-H489A Reverse	CACCTGTATGCGTTCTGAGGGCAATATTGAGGTAATAATTTG	N/A
V5-ZNF217_pLX307	N/A	Purchased from Addgene, plasmid #98384[1]	N/A

Table S8. List of primers used for molecular cloning.

Full Unedited, Annotated Blots
Main Figures

ZNF217 promotes ovarian cancer progression by impacting multiple pivotal steps in the metastatic process

Wardrup KC et al.

Full Unedited Blots: Figure 1C

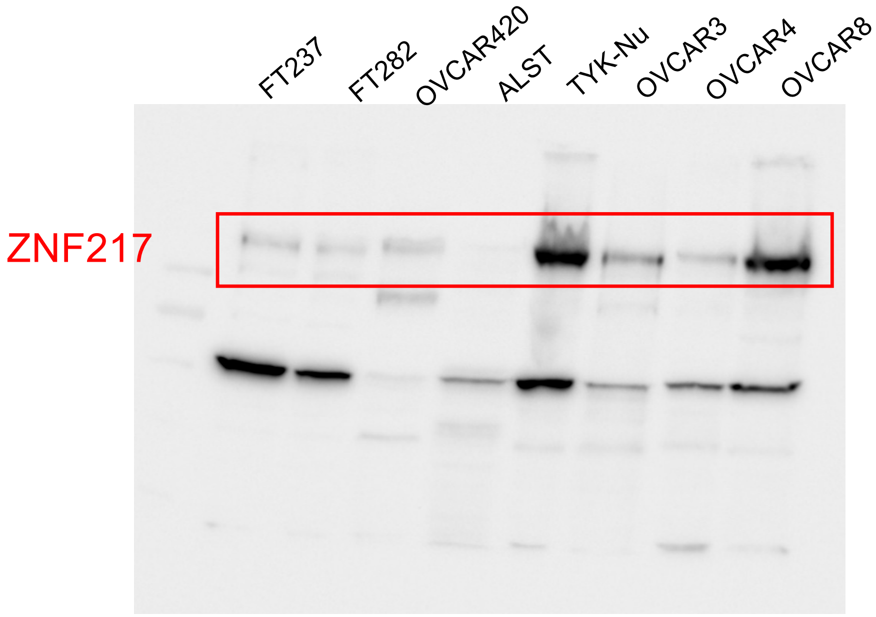

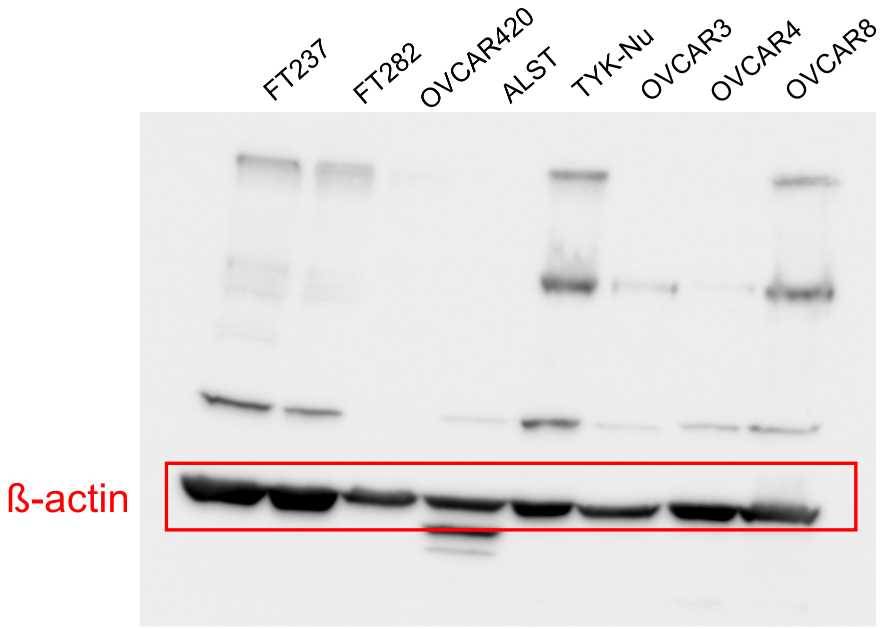

Full Unedited Blots: Figure 2A

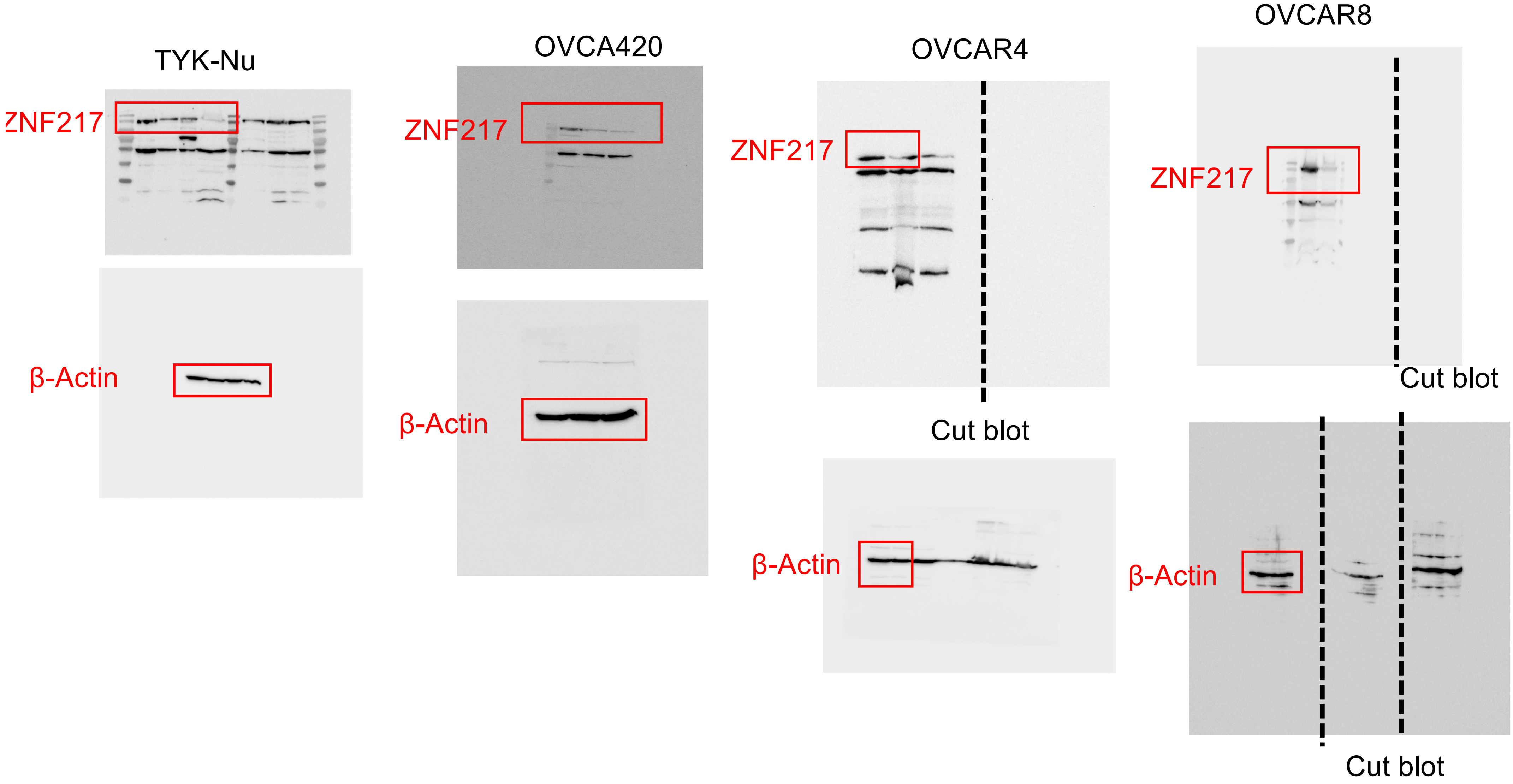

Full Unedited Blots: Figure 2A (Cont).

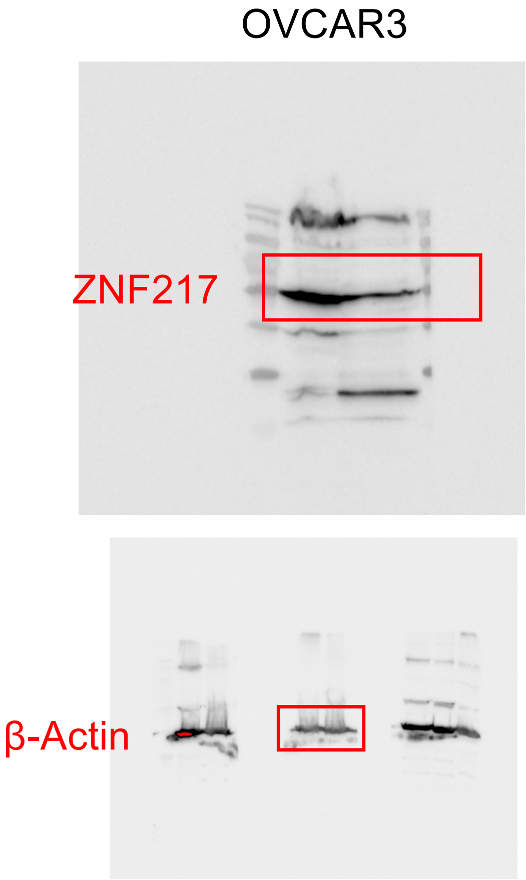

Full Unedited Blots: Figure 2G

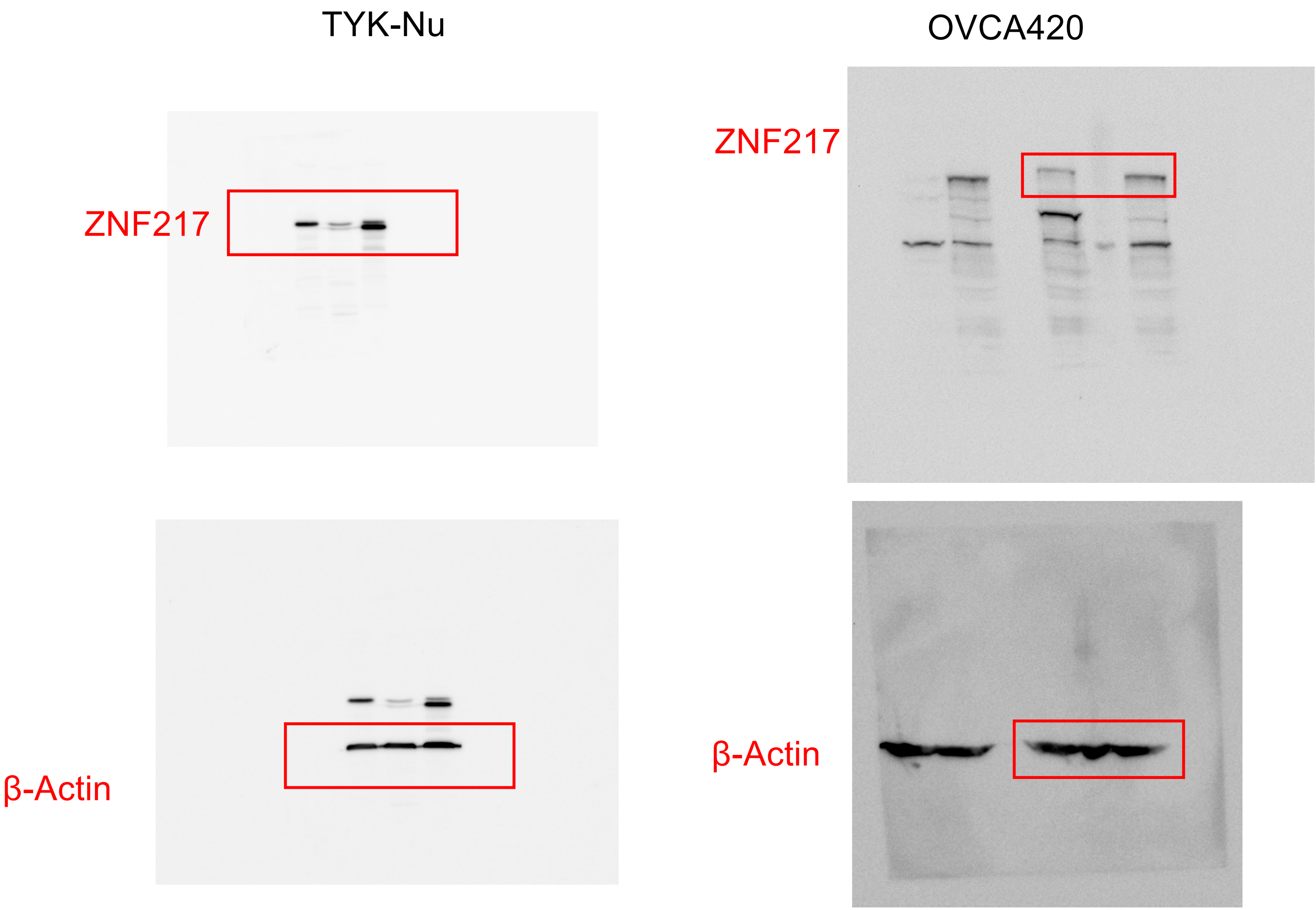

Full Unedited Blots: Figure 3B

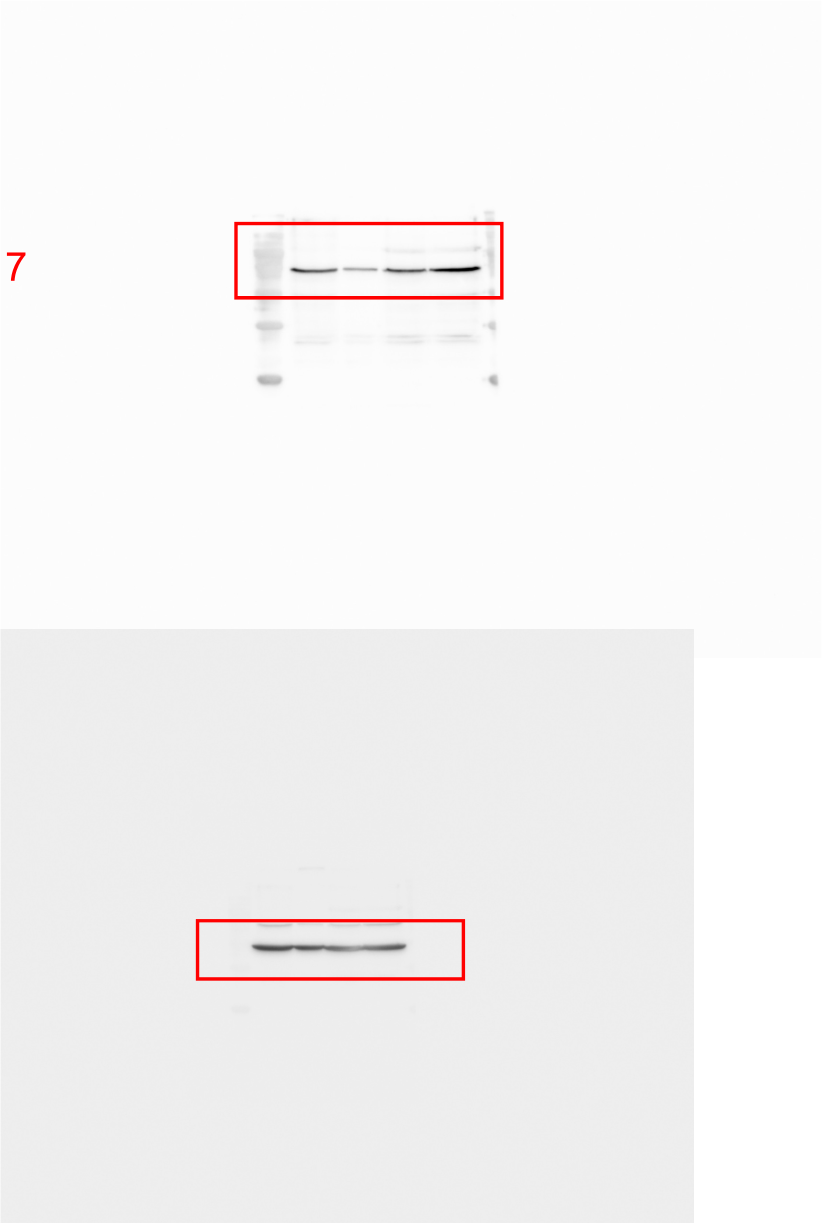

Full Unedited Blots: Figure 3G

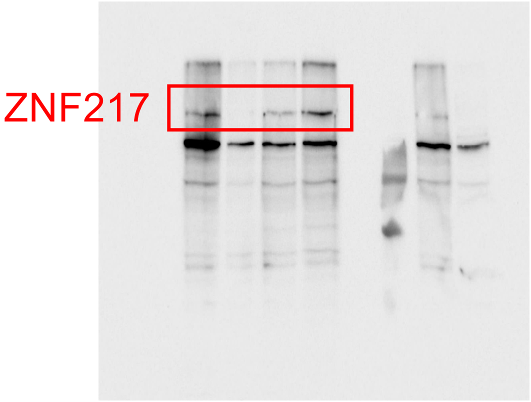

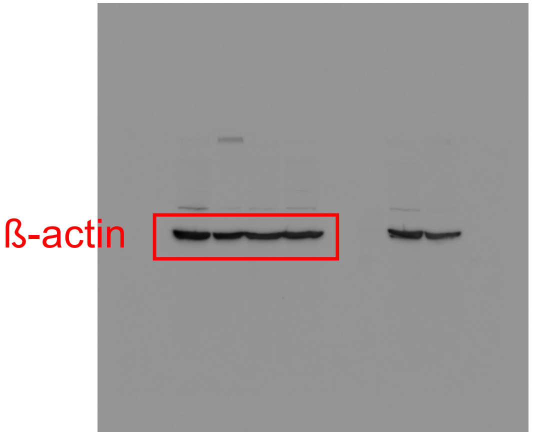

Full Unedited Blots: Figure 4H

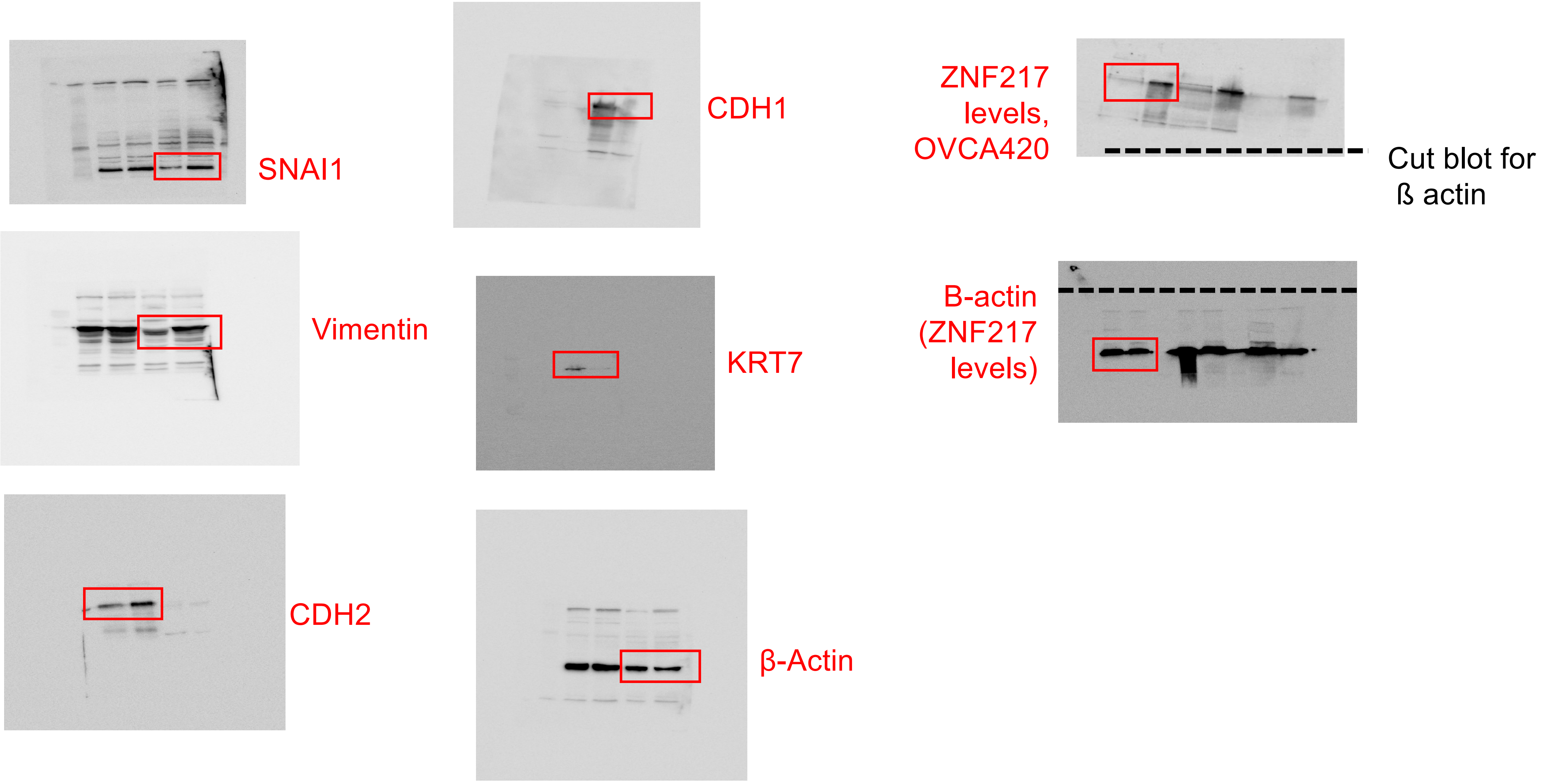

Full Unedited Blots: Figure 4I

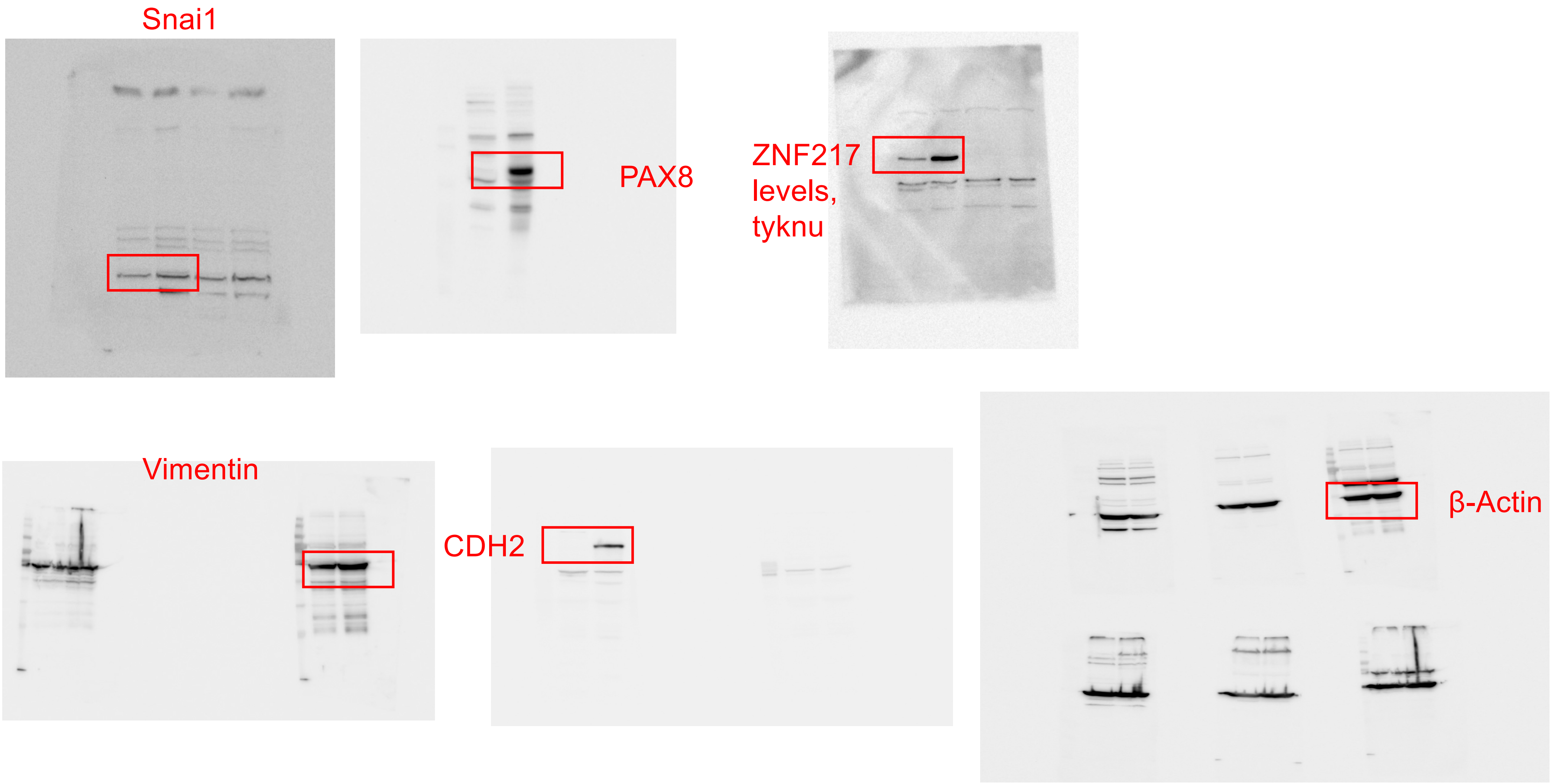

Full Unedited Blots: Figure 5A

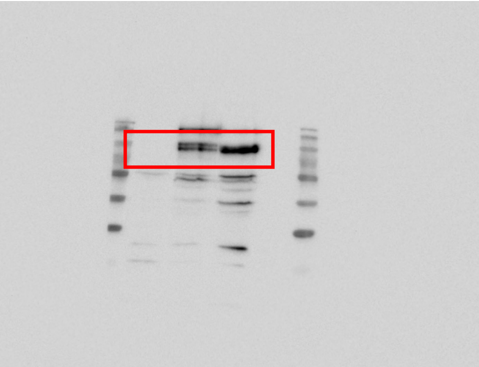

FLAG

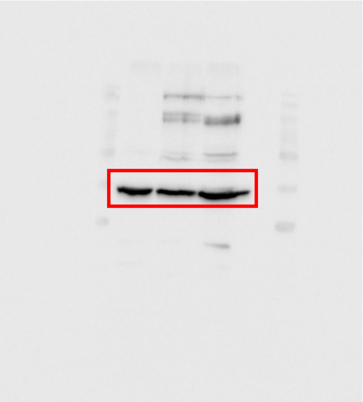

β-Actin

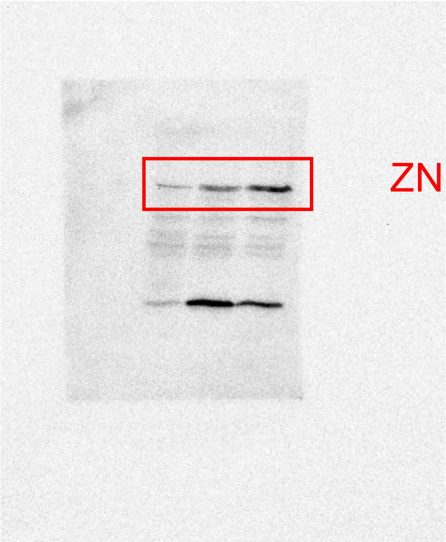

ZNF217

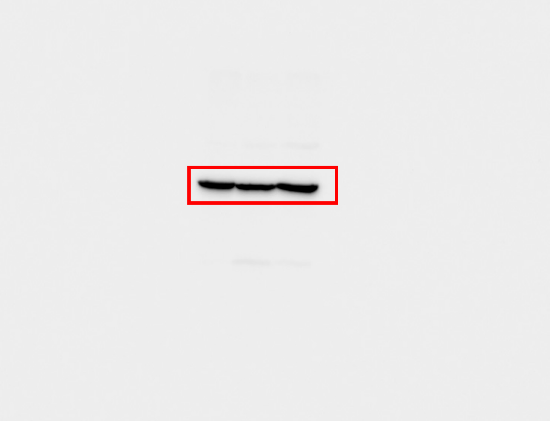

β-Actin

Full Unedited Blots: Figure 6A

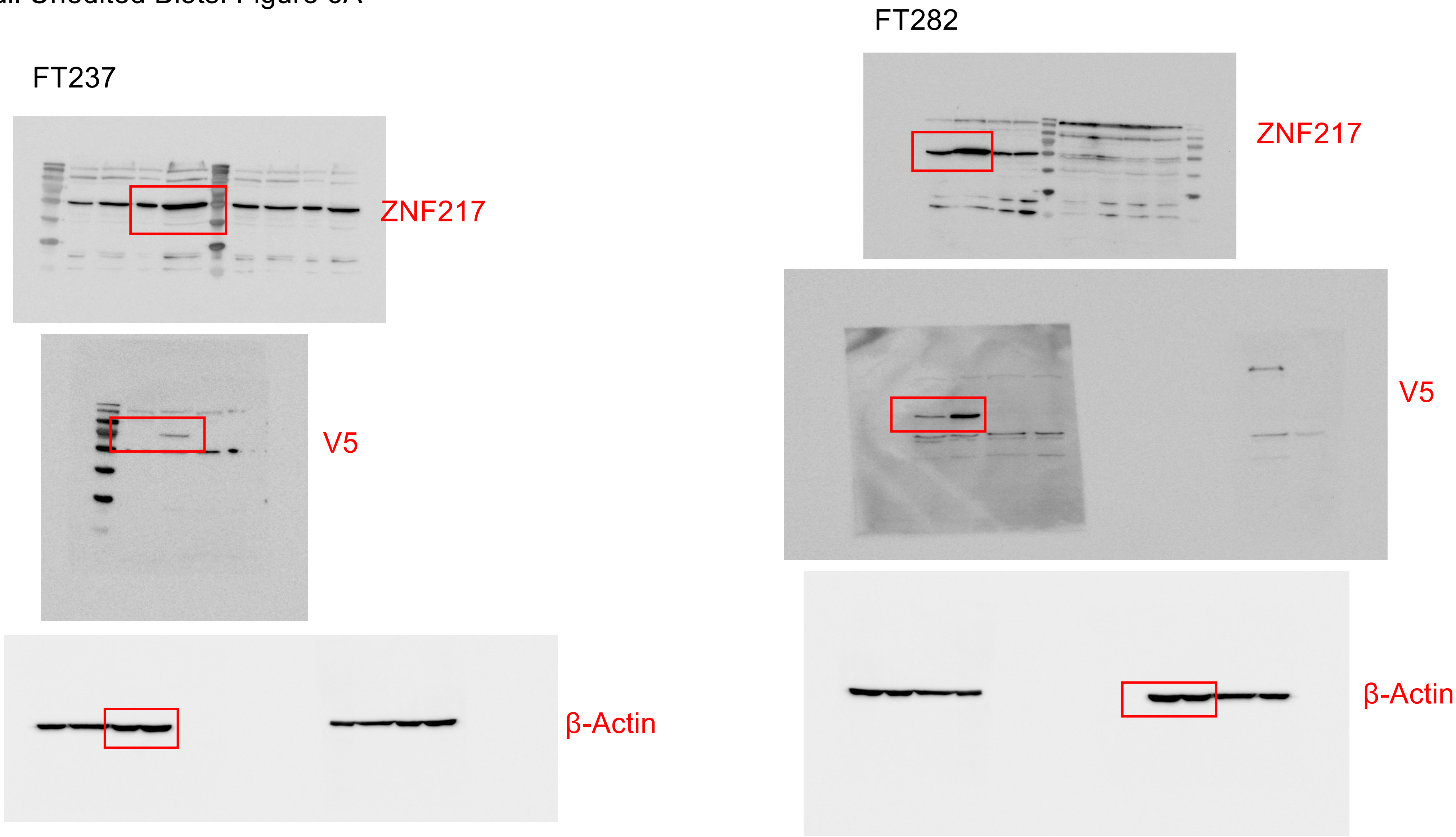

Full Unedited Blots: Figure 6F

FT237

SNAI1

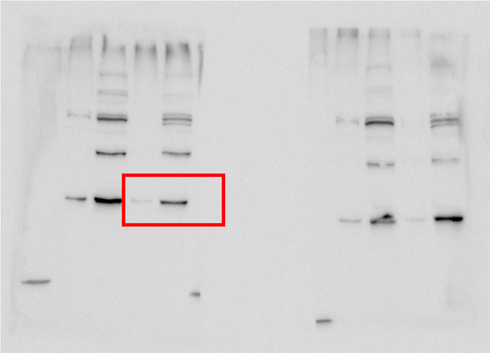

VCAM1

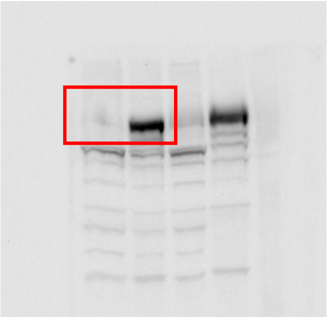

CDH2

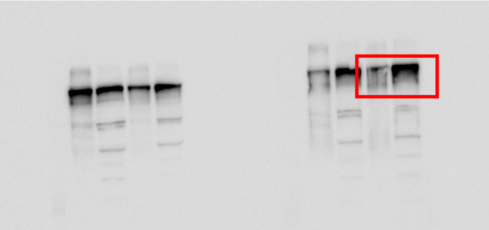

ZNF217 levels

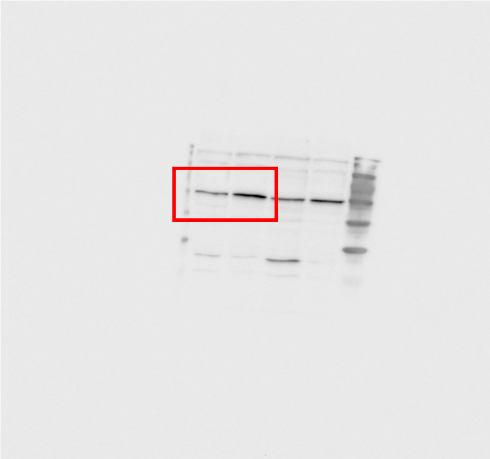

ZEB1

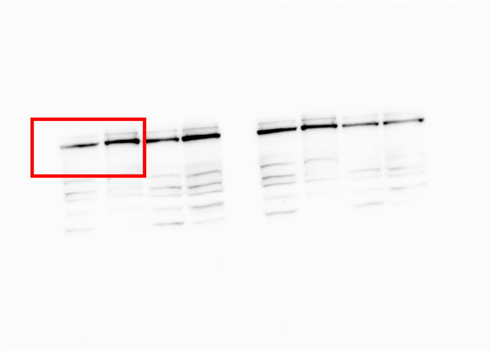

β-Actin

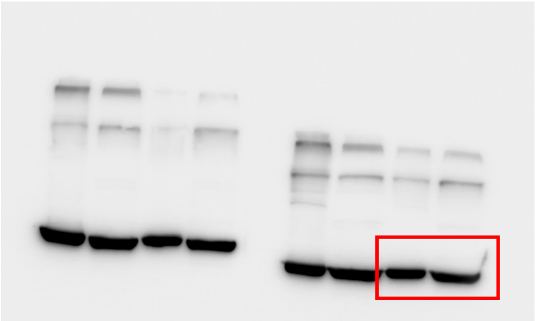

Full Unedited Blots: Figure 6F

FT282

SNAI1

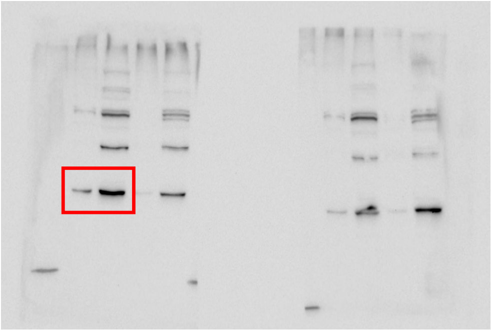

VCAM1

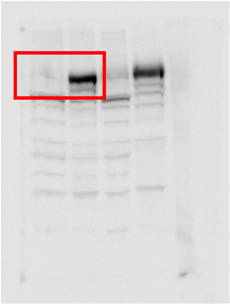

CDH2

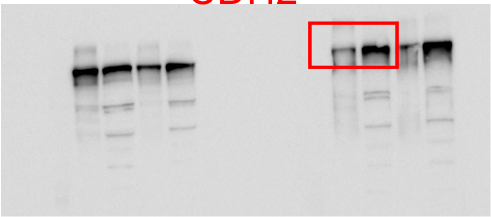

ZEB1

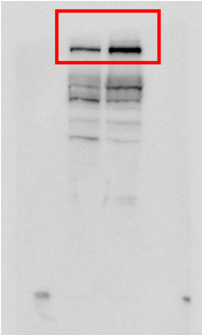

β-Actin

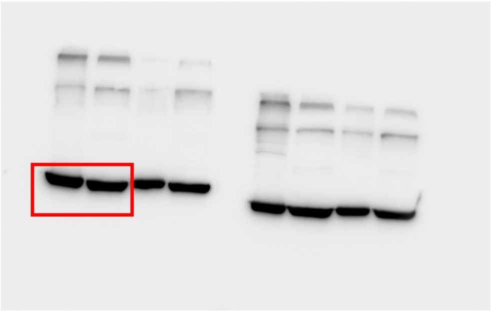

Full Unedited, Annotated Blots
Supplemental Figures

ZNF217 promotes ovarian cancer progression by impacting multiple pivotal steps in the metastatic process

Wardrup KC et al.

Full Unedited Blots: Figure S1C

OVCA420 cells

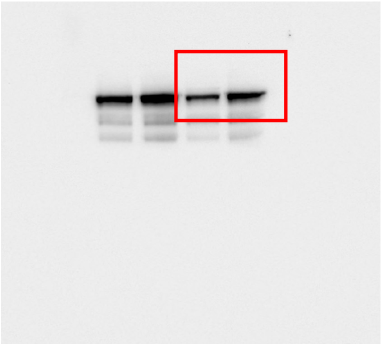

ZNF217

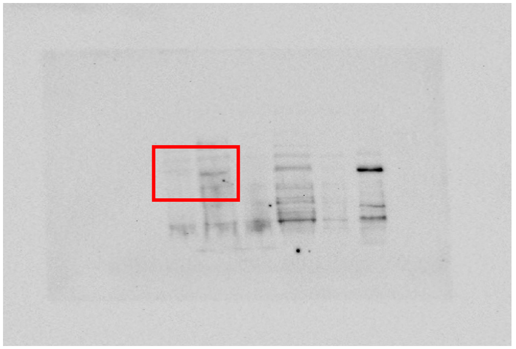

FLAG

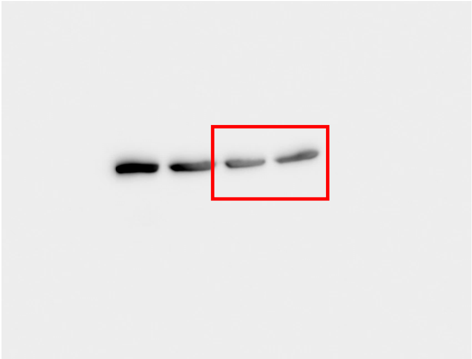

β-Actin

Full Unedited Blots: Figure S1C

TYK-Nu cells

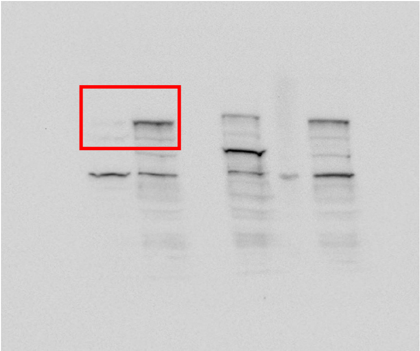

ZNF217

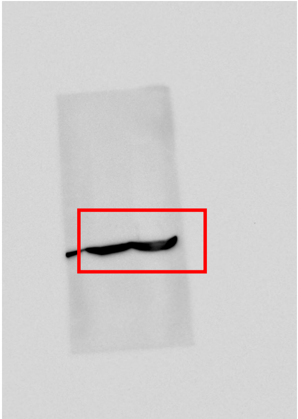

β-Actin

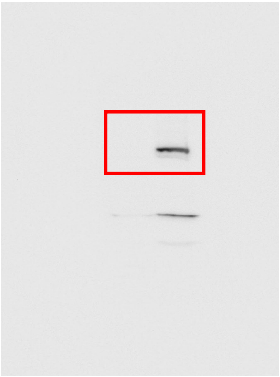

V5

Full Unedited Blots: Figure S1C

ALST cells

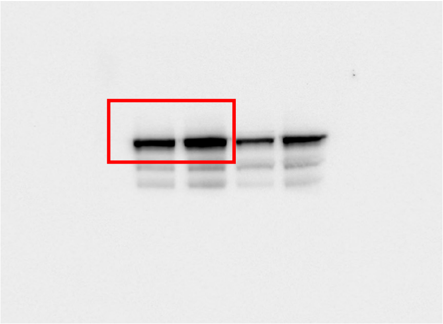

ZNF217

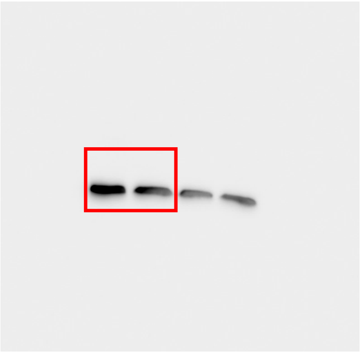

β-Actin

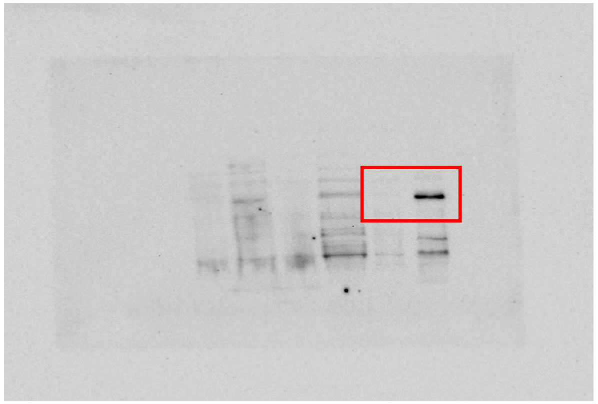

FLAG
